# Supplementary material for: Trunk rotation, spinal deformity and appearance, health-related quality of life, and treatment adherence: Secondary outcomes in a randomized controlled trial on conservative treatment for adolescent idiopathic scoliosis
Source: PLoS One. 2025 Apr 21;20(4):e0320581. doi: 10.1371/journal.pone.0320581 (PMC12011275; doi:10.1371/journal.pone.0320581)
Supplement: S2 File — (DOCX) [file pone.0320581.s002.docx]

| Section/Topic | Item | CONSORT 2010 Statement Checklist Item, PRO-specific Extensions Are Prefaced by the letter P [1,2] | | Reported on page No |
| --- | --- | --- | --- | --- |
| Title and abstract | | | | |
|  | 1a | Identification as a randomised trial in the title | | Title page |
|  | 1b | Structured summary of trial design, methods, results, and conclusions (for specific guidance see CONSORT for abstracts)  *P1b: Patient reported outcomes (PRO) are identified in the abstract as secondary outcome* | | 3-4 |
| Introduction | | | | |
| Background and objectives | 2a | Scientific background and explanation of rationale | | 4-5 |
|  | 2b | Specific objectives or hypotheses, *P2b: Explorative study with PRO´s stated* | | 5 |
| Methods | | | | |
| Trial design | 3a | Description of trial design (such as parallel, factorial) including allocation ratio | | 5 |
|  | 3b | Important changes to methods after trial commencement (such as eligibility criteria), with reasons | | Not applicable (NA) |
| Participants | 4a | Eligibility criteria for participants | | 6 |
|  | 4b | Settings and locations where the data were collected | | 6 |
| Interventions | 5 | The interventions for each group with sufficient details to allow replication, including how and when they were actually administered | | 6-7 |
| Outcomes | 6a | Completely defined pre-specified primary and secondary outcome measures, including how and when they were assessed. *P6a: PRO instrument validity and reliability are cited and method includes data collection* | | 7-10 |
|  | 6b | Any changes to trial outcomes after the trial commenced, with reasons. | | NA |
| Sample size | 7a | How sample size was determined | | 10 |
|  | 7b | When applicable, explanation of any interim analyses and stopping guidelines.  *In case of treatment failure* | | NA |
| Randomisation: |  |  | |  |
| Sequence generation | 8a | Method used to generate the random allocation sequence | | 10 |
|  | 8b | Type of randomisation; details of any restriction (such as blocking and block size) | | 10 |
| Allocation concealment mechanism | 9 | Mechanism used to implement the random allocation sequence (such as sequentially numbered containers), describing any steps taken to conceal the sequence until interventions were assigned | | 10 |
| Implementation | 10 | Who generated the random allocation sequence, who enrolled participants, and who assigned participants to interventions. | | 10 |
| Blinding | 11a | If done, who was blinded after assignment to interventions (for example, participants, care providers, those assessing outcomes) and how | | 10 |
|  | 11b | If relevant, description of the similarity of interventions | | 6 |
| Statistical methods | 12a | Statistical methods used to compare groups for primary and secondary outcomes. *P12a: Statistical approaches dealing with missing data are explicitly stated* | | 11-12 |
|  | 12b | Methods for additional analyses, such as subgroup analyses and adjusted analyses. *Sensitivity analyses.* | | 12 |
| Results | | | | |
| Participant flow (a diagram is strongly recommended) | 13a | For each group, the numbers of participants who were randomly assigned, received intended treatment, and were analysed for the primary outcome | | 10 CONSORT flow diagram |
|  | 13b | For each group, losses and exclusions after randomisation, together with reasons | | 10 CONSORT flow diagram |
| Recruitment | 14a | Dates defining the periods of recruitment and follow-up | | 6 |
|  | 14b | Why the trial ended or was stopped | | NA |
| Baseline data | 15 | A table showing baseline demographic and clinical characteristics for each group | | Table 1 |
| Numbers analysed | 16 | For each group, number of participants (denominator) included in each analysis and whether the analysis was by original assigned groups. *Figure 1 CONSORT flow diagram, Intention to treat analyses or per protocol as appropriate.* | | 10 |
| Outcomes and estimation | 17a | For each primary and secondary outcome, results for each group, and the estimated effect size and its precision (such as 95% confidence interval) | | 15-23  Table 2-6 |
|  | 17b | For binary outcomes, presentation of both absolute and relative effect sizes is recommended | | NA |
| Ancillary analyses | 18 | Results of any other analyses performed, including subgroup analyses and adjusted analyses, distinguishing pre-specified from exploratory | | 24 |
| Harms | 19 | All important harms or unintended effects in each group (for specific guidance see CONSORT for harms) | | NA |
| Discussion | | | | |
| Limitations | 20 | Trial limitations, addressing sources of potential bias, imprecision, and, if relevant, multiplicity of analyses  P20: PRO-specific limitations are discussed | | 26-27 |
| Generalisability | 21 | Generalisability (external validity, applicability) of the trial findings  *P21: PRO-specific implications för generalizability are discussed* | | 25-26 |
| Interpretation | 22 | Interpretation consistent with results, balancing benefits and harms, and considering other relevant evidence | | 24-25 |
| Other information | | | |  |
| Registration | 23 | Registration number and name of trial registry | | 5 |
| Protocol | 24 | Where the full trial protocol can be accessed, if available | | 5 |
| Funding | 25 | Sources of funding and other support (such as supply of drugs), role of funders | Submission process information | |

References

1. Moher, D.; Hopewell, S.; Schulz, K.F.; Montori, V.; Gotzsche, P.C.; Devereaux, P.J.; Elbourne, D.; Egger, M.; Altman, D.G.; Consort. CONSORT 2010 explanation and elaboration: updated guidelines for reporting parallel group randomised trials. *Int J Surg* **2012**, *10*, 28-55, doi:10.1016/j.ijsu.2011.10.001.

2. Calvert, M.; Blazeby, J.; Altman, D.G.; Revicki, D.A.; Moher, D.; Brundage, M.D. Reporting of patient-reported outcomes in randomized trials: The CONSORT PRO extension. *JAMA: Journal of the American Medical Association* **2013**, *309*, 814-822, doi:10.1001/jama.2013.879.
