# Supplementary material for: Trunk rotation, spinal deformity and appearance, health-related quality of life, and treatment adherence: Secondary outcomes in a randomized controlled trial on conservative treatment for adolescent idiopathic scoliosis
Source: PLoS One. 2025 Apr 21;20(4):e0320581. doi: 10.1371/journal.pone.0320581 (PMC12011275; doi:10.1371/journal.pone.0320581)
Supplement: S3 File — (PDF) [file pone.0320581.s003.pdf]

<u>**Ethics application documents**</u>

Initial application form to the Swedish ethical review authority Dnr 2012/172-31/4 (In Swedish) – Pages 2-19

Project plan version 1.1 submitted in the initial application to the Swedish ethical review authority Dnr 2012/172-31/4 **(In English)** – Pages 20-29

Annexes to the initial application form (In Swedish) – Pages 30-74

Swedish ethical review authority decision of approval letter for the initial application Dnr 2012/172-31/4 (In Swedish) – Pages 75-76

Swedish ethical review authority decision of approval letter for the initial application Dnr 2012/172-31/4 **(In English)** – Pages 77-78

Swedish ethical review authority decision of approval letter for the amendment Dnr 2015/1007-32 (In Swedish) – Page 79

Swedish ethical review authority decision of approval letter for the amendment Dnr 2015/1007-32 **(In English)** – Page 80

Swedish ethical review authority decision of approval letter for the amendment Dnr 2017/609-32 (In Swedish) – Page 81

Swedish ethical review authority decision of approval letter for the amendment Dnr 2017/609-32 **(In English)** – Page 82

ANSÖKAN OM ETIKPRÖVNING

Information till ansökan, ***se bilaga och Vägledningar*** *(www.epn.se)*

Till Regionala etikprövningsnämnden i: Stockholm

Den regionala etikprövningsnämnd till vars upptagningsområde forskningshuvudmannen hör, se respektive nämnd *(www.epn.se)*

Avgift inbetald datum: 120123

Observera att en ansökan aldrig är komplett och därmed kan behandlas förrän blanketten är korrekt ifylld och avgiften är betald.

Projekttitel: Skolios- resultat av behandling

Ange en beskrivande titel på svenska för lekmän, utan sekretesskyddad information. Ange också i förekommande fall projektets identitet, projektets/forskningsplanens (protokollets eller prövningsplanens) nummer, version, datum osv.

Projektnummer/identitet: Version nummer: 1

Uppgifter som fylls i av den regionala etikprövningsnämnden

Ansökan komplett: Dnr:

Begäran om ytterligare information (i sak): Begärd information inkommen:

Beslutsdatum: Expeditionsdatum:

Ansökan avser (gäller även vid begäran om rådgivande yttrande):

Forskning där endast en forskningshuvudman deltar (5 000 kr)	☒
Forskning där mer än en huvudman deltar (16 000 kr)	☐
Forskning där mer än en forskningshuvudman deltar, men där samtliga forskningspersoner eller forskningsobjekt har ett omedelbart samband med endast en av forskningshuvudmännen (5 000 kr)	☐
Endast behandling av personuppgifter (5 000 kr)	☐
Forskning som gäller klinisk läkemedelsprövning (16 000 kr)	☐
Ändring av tidigare godkänd ansökan enligt 4 § förordning (2003:615) om etikprövning av forskning som avser människor (2 000 kr)	☐

Om nämnden finner att forskningsprojektet inte faller inom etikprövninglagens tillämpningsområde önskas ett rådgivande yttrande. (Info: 4a och 4b §§ i förordning 2003:615) (Info: Bilaga till ansökan)

Ja: ☒ Nej: ☐

1. Information om forskningshuvudman m.m.

1:1 Forskningshuvudman (Info: p. 1:1 i Vägledning till ansökan)

Ansökan om etikprövning av forskning ska göras av forskningshuvudmannen. *Med forskningshuvudman avses en statlig myndighet eller en fysisk eller juridisk person i vars verksamhet forskningen utförs.* Inom staten utförs forskning främst vid lärosätena, men även vid vissa andra myndigheter, som t.ex. Brottsförebyggande rådet och Socialstyrelsen. Kommuner och landsting kan vara forskningshuvudmän, liksom privaträttsliga juridiska personer.

Namn: Stockholms läns landsting

Adress: Box 22500, 104 22 Stockholm

1:2 Behörig företrädare för forskningshuvudmannen

Behörig företrädare är t.ex. prefekt, enhetschef, verksamhetschef. Forskningshuvudmännen bestämmer själva, genom interna arbets- och delegationsordningar eller genom fullmakt, vem som är behörig att företräda forskningshuvudmannen. Kopia av sådan handling *ska* bifogas.

Namn: Lennart Adamsson Tjänstetitel: Verksamhetschef

Adress: Ortopedkliniken, K54, Karolinska Universitetssjukhuset Huddinge

1:3 Forskare som är huvudansvarig för genomförandet av projektet (kontaktperson)

(Info: p. 1:3 i Vägledning till ansökan)

Namn: Paul Gerdhem Tjänstetitel: Docent, överläkare

Adress: Ortopedkliniken, K54, Karolinska Universitetssjukhuset Huddinge

E-postadress: paul.gerdhem@karolinska.se

Telefon: 08-58580000

Mobiltelefon: 0736-994409

1:4 Plats (Info: p. 1:4 i Vägledning till ansökan)

Plats (er) där projektet ska genomföras, ange inrättning (ar), institution (er), klinik (er) etc.

Ortopedkliniken, Karolinska Universitetssjukhuset, Huddinge

1:5 Andra medverkande

Övriga deltagande forskningshuvudmän samt forskare ansvariga för att lokalt genomföra projektet (kontaktpersoner) anges här eller i bilaga med namn och adresser (se p. 9 bilaga nr 1).

Allan Abbott, Sjukgymnastkliniken, Karolinska Universitetssjukhuset, Huddinge, 141 86 Stockholm (Stockholms läns landsting)

Tel: 08-58580000

Övriga medverkande forskare: Kourosh Jalalpour, Hans Möller, Helena Normelli, Helena Saraste, Tomas Reigo; samtliga vid ortopedkliniken, K54, Karolinska Universitetssjukhuset, Huddinge. Tel 08-58580000.

Christos Topalis, barnortopediska kliniken, Karolinska Universitetssjukhuset Solna. Tel: 08-51770000

1:6 Ansökan/anmälan till andra myndigheter

Vid läkemedelsprövning

Ansökan om tillstånd av *Läkemedelsverket* – se Läkemedelsverkets hemsida (www.mpa.se)

Ansökan inlämnad (datum)

Tillstånd erhållits ☐

EudraCT nr:

Vid viss genetisk forskning

Om personuppgifter om genetiska anlag som har framkommit efter genetisk undersökning kommer att hanteras i studien ska detta anmälas till *Datainspektionen* enligt 10 § personuppgiftsförordningen (1998:1191) – se Datainspektionens hemsida www.datainspektionen.se

Anmälan inlämnad (datum) Kommer att inlämnas efter godkänd etikprövning ☐

Vid viss forskning som innefattar bestrålning av forskningspersoner (Info: p. 9 i Vägledning till ansökan)

Ansökan, enligt 16 och 22 §§ Strålsäkerhetsmyndighetens föreskrifter (SSMFS 2008:35) om allmänna skyldigheter vid medicinsk och odontologisk verksamhet med joniserande strålning, till *Strålskyddskommitté* – för vidare information kontakta aktuell lokal strålskyddskommitté.

Ansökan inlämnad (datum): 120120 Ansökan tillstyrkt ☐

2. Uppgifter om projektet

2:1 Sammanfattande beskrivning av forskningsprojektet (programmet)

Vägledning till forskningsplan/forskningsprotokoll (program) (Info: p. 9 i Vägledning till ansökan)

Beskrivningen ska kunna förstås av nämndens samtliga ledamöter. Undvik därför terminologi som kräver specialkunskaper. Ange bakgrund och syfte för studien samt den/de vetenskapliga frågeställning (ar) som man söker svar på. Ange de viktigaste undersökningsvariablerna. Beskriv vilka kunskapsvinster projektet kan förväntas ge och betydelsen av dessa. Ange om det är en registerstudie, uppdragsforskning etc. För fackmän avsedd detaljerad information i forskningsplan/forskningsprotokoll (program) _ska_ bifogas som bilaga (se p. 9 bilaga nr 2). En utförligare beskrivning av studiens genomförande *avsedd för lekmän* kan vid behov bifogas den för fackmän avsedda obligatoriska forskningsplanen.

Idiopatisk skolios är den vanligaste formen av ryggradskrökning. Uttalad skolios kan leda till ryggbesvär och lungpåverkan, utöver den psykiska påverkan som ryggdeformiteten kan ge. Skolios drabbar ca 3% av barn och ungdomar. De flesta som får skolios hittas inom skolhälsovården där screening utförs. Dessa remitteras för närmare diagnostik till ortoped. Cirka en tiondel av de som drabbas får en mer uttalad skolios som behandlas med korsett eller operation. Detta projekt består av fyra delstudier med huvudsyfte att förbättra behandlingen för personer med skolios.

Delstudie 1.

Bakgrund: Randomiserade studier av effekten av korsettbehandling saknas idag. Randomiserade studier av effekten av träning på skolios saknas idag.

Frågeställning: Kan progress av idiopatisk skolios förhindras med behandling av nattkorsett eller med hållningsträning jämfört med konditionsträning?

Metod: Randomiserad kontrollerad studie. Randomisering till behandling med nattkorsett, hållningsträning eller konditionsträning.

Deltagare: Barn med minst ett års längdtillväxt kvar. Behandlingstid uppskattas till 2-5 år. Forskningspersonerna kommer att lottas till behandling med nattkorsett eller ett av två träningsprogram. Vi beräknar att totalt 135 barn behöver ingå i studien. Forskningspersonerna kommer att följas från inklusion i upp till ca tio år efter avslutad tillväxt. De som inte vill delta i studien kommer att användas för jämförelser. Journal och röntgendata kommer att inhämtas från dessa.

Viktigaste undersökningsvariabel: Misslyckad behandling. Misslyckad behandling definieras som en röntgenologisk ökning av skoliosen mer än 6 grader, mätt enligt Cobb, jämfört med röntgenbilden vid inklusion. Ökningen skall ha konstaterats på två på varandra följande

röntgenundersökningar. Personer där skoliosen ökar mer än 6 grader kommer att erbjudas behandling med sk Bostonkorsett (behandlingstid ca 20 timmar per dygn).

Kunskapsvinster: Trots att korsettbehandling utförts i decennier saknas än idag evidens för effekt från randomiserade studier. Den högsta evidensgrad som finns kommer ifrån en prospektiv kontrollerad studie som visade effekt av användande av en korsett dygnet runt (egentligen mer än 20 timmar per dygn) jämfört med enbart observation. Denna studie har blivit kritiserad. Övriga studier är observationsstudier utan kontrollgrupp. Träning förespåkas av en del som en möjlig behandling. Randomiserade studier saknas.

Det är idag ofta svårt att få barn att acceptera användning av korsett dygnet runt. Användande av korsett enbart nattetid, som ger en större korrektion av skolioskröken vid användning jämfört med en dygnetrunt-korsett, har lanserats som ett alternativ. En stor fördel är att en korsett som enbart används nattetid är enklare att fördra. Preliminära data antyder att en nattkorsett inte ger sämre resultat än användande av en korsett dygnet runt.

Screeningverksamhet av skolios bygger på att korsettbehandling har effekt, men underlaget för att bedöma detta är idag otillräckligt då evidens för effekten av korsettbehandling är svag.

Delstudie 2.

Bakgrund: Information om resultat av operation vid olika typer av skolios och olika typer av operationer på nationell nivå finns inte idag.

Frågeställning: Hur mår patienter efter operation av skolios? Skiljer sig resultaten beroende på ålder vid operation och orsak till skoliosen?

Metod: Registerstudie.

Patienter som opereras för skolios i Sverige registreras sedan 2007 i det svenska ryggregistret. Tillgängliga data består av information om typ av skolios, utförd operation och livskvalité före och efter operation mätt med de validerade frågeformulären EQ5D, SRS22 och basdata avseende bla längd, vikt och grad av smärta. Tillstånd för uthämtning av data finns från styrelsen för Svensk ryggkirurgisk förening som är registeransvarig. Fn finns ca 1300 operationer införda i registret. Operationsberättelser och röntgenbilder innan och efter operation som är del av den normala sjukvården kommer att inhämtas från behandlande sjukhus för validering av data som införts i registret. För att bedöma registrets representativitet avseende diagnoser, utförda operationer och komplikationer kommer en jämförelse med socialstyrelsens öppen- och slutenvårdsregister avseende skoliosdiagnoser, skoliosoperationer och komplikationer utföras. Patienterna kommer inte att kontaktas.

Viktigaste undersökningsvariabel: Frågeformuläret SRS22 omräknat till ett speciellt index.

Kunskapsvinst: Information om livskvalité efter operation av skolios på nationell bas saknas idag och denna kunskapslucka kommer att fyllas av denna studie. Information om typ av skolios som leder till operation på nationell nivå saknas idag.

Delstudie 3.

Bakgrund: Bestämning av skelettåldern är idag det bästa sättet att förutspå en skolioskröks potential att öka och är ett viktigt kliniskt hjälpmedel. Idag bestäms skelettåldern med hjälp av en röntgenundersökning av handen, armbågen eller bäckenet.

Metod: Kross-sektionell observationsstudie hos fall och kontroller.

BonAge är en apparat som använder ultraljud för att bestämma skelettålder. Om ultraljud kan användas istället kan användandet av röntgen och därmed stråldosen minskas hos barn med skolios. Vi använder redan denna teknik i en annan studie ("Livskvalitet och biol markörer vid skolios" Dnr 2009/696 31/2) men önskar nu undersöka fler barn. Undersökningen tar ca fem minuter och innebär att en ultraljudsdosa sätts mot handleden.

Undersökningen är smärtfri och ofarlig. Vi önskar utföra denna undersökning på barn som rutinmässigt undersöks för skolios på vår mottagning. Dessutom önskar vi utföra denna undersökning på barn som av andra skäl besöker ortopedmottagningen eller barnortopedmottagningen på Karolinska Universitetssjukhuset.

Vi planerar att undersöka ca 150 barn med skolios och 150 barn som undersöks av andra orsaker.

Viktigaste undersökningsvariabel: Skelettålder mätt med ultraljud som kommer att jämföras med kronologisk ålder.

Kunskapsvinst: Data från denna studie och en annan pågående studie kan ge information om skelettålder kan bestämmas med ultraljud. Om detta är en användbar metod skulle det kunna vara en hjälp vid behandlingen av patienter med skolios, men också kunna vara viktig vid andra tillfällen där man idag bestämmer skelettåldern med röntgenteknik.

Delstudie 4.

Bakgrund: Ett av de idag mest använda frågeformulären för bestämning av livskvalité hos skoliospatienter är SRS22. Frågeformuläret har utvecklats av Scoliosis Research Society men hittills har bara en enda studie där formuläret använts hos icke-skoliotiska personer rapporterats. SRS22 används i det svenska ryggregistret och kommer också att vara en del av delstudie 1 och 3 ovan.

Frågeställning: Hur besvaras SRS22 av en normalpopulation?

Metod: Kross-sektionell observationsstudie. Enkätstudie. SRS22 skickas per brev tillsammans med andra frågor som tidigare använts i våra egna studier av skoliospatienter. Vi önskar svar från 500 slumpmässigt utvalda personer fördelat på olika åldersgrupper. Personerna väljs ur befolkningsregistret och kommer att kontaktas per brev. Om svar inte erhålls kommer vi att försöka få kontakt per telefon.

Viktigaste undersökningsvariabel: SRS22 omräknat till ett speciellt index.

Kunskapsvinst: Normaldata för en svensk befolkningen av SRS22 kommer att fås. Detta är värdefullt för ovan beskrivna delstudier och dessutom för redan avslutade studier av skoliospatienter.

2:2 Vilken/vilka vetenskaplig (a) frågeställning (ar) ligger till grund för projektets utformning?

Om projektet kan karakteriseras som en hypotesprövning, ange den primära och eventuellt sekundära hypotesen. Hänvisning till mer detaljerad information för fackmän kan ske till bifogad forskningsplan enligt punkt 2:1

Är användande av nattkorsett eller träning ett effektivt sätt att förhindra ökning av skolios?

Hur förändras livskvalitén efter skolioskirurgi?

Är skelettåldersbestämning med ultraljud relaterat till kronologisk ålder hos barn med och utan skolios?

Hur besvarar en population ur befolkningen det skoliosspecifika livskvalitéformuläret SRS22?

2:3 Redogör för resultat från relevanta djurförsök

Om djurförsök inte utförts ange skälen till detta.

Ej relevant. Djurmodeller för studie av korsettbehandling och resultat av operation för skolios saknas.

2:4 Redogör översiktligt för undersökningsprocedur, datainsamling och datas karaktär

(Info: p. 2:4 i Vägledning till ansökan)

Av beskrivningen ska framgå hur projektet planeras genomföras. Beskriv insamlade datas karaktär. Ange hur datas tillförlitlighet säkerställs (t.ex. kvalitetskontroll/monitorering). - Vid enkäter och intervjuer ska beskrivas tillvägagångssätt och t.ex. frågors innehåll och hur slutsatser dras. Enkäter och skattningsskalor *ska* bifogas (se p. 9 bilaga nr 5). - För medicinsk forskning ska anges t.ex. typer av ingrepp, mätmetoder, antal besök, tidsåtgång vid varje försök, doser och administrationssätt för eventuella läkemedel och/eller isotoper, blodprovsmängd

(även ackumulerad mängd vid multipla försök). Ange även om och på vilket sätt undersökningsprocedur m.m. skiljer sig från klinisk rutin. Ange proceduren för att ge den eventuella behandling efter projektets slut, som kan erfordras. Ange procedur för insamling av biologiskt material. Redogör för datakällor och procedurer vid behandling av personuppgifter. För mer detaljerad information kan hänvisning ske till bilagd forskningsplan.

Delstudie 1

Undersökningsprocedur: Personer med skolios som undersöks vid ortopedkliniken och uppfyller inklusionskriterierna tillfrågas om deltagande. Deltagande personer följs var 6:e månad tills tillväxten avslutats. Detta motsvarar dagens kliniska rutin för korsettbehandlade personer. Tidsåtgången per besök uppskattas till ca 60 min. Tidsåtgången uppskattas öka med 20 min jämfört med ett rutinbesök, vilket motsvarar tiden för att fylla i frågeformulären. Behandlingen avslutas då patientens tillväxt är avslutad. Efter avslutad behandling kommer forskningspersonen att erbjudas uppföljning upp till 10 år efter avslutad behandling. Preliminärt kommer uppföljning att ske efter ca 2 år, 5 år och 10 år. Se även bifogat forskningsprogram.

Korsettgruppen: Erhåller speciellt anpassad nattkorsett via ortopedingenjör. Denna bärs 8 timmar per natt. Utöver korsettbehandlingen uppmanas patienterna utföra ett hemträningsprogram för att uppnå minst 60 minuters fysisk aktivitet dagligen.

Hållningsträningsgrupp: Ett besök per månad hos sjukgymnast under de 3 första månaderna efter inklusion (totalt 3 besök). Gruppen får instruktioner att utföra ett hållningsträningsprogram på ca 30 minuter dagligen och utöver detta minst 30 minuters konditionsträning.

Konditionsträningsgrupp: Gruppen får instruktioner att utföra minst 60 minuters fysisk aktivitet dagligen.

Datainsamling och datas karaktär: Frågeformulär används för att samla information om personnummer, namn, hälsotillstånd och andra basdata. Frågeformulären EQ5D-Y, SRS22r, SAQ och IPAQ används. Samtliga är validerade frågeformulär och utvärderas på standardiserat sätt. Röntgeninformation från den röntgenbild som tas för att diagnosticera skolios sparas: Cobbvinklar, apex- och ändkotor, klassificering av krök enl King och Lenke. Röntgenbilden sparas. Information om korsetten sparas.

Informationen förs in i en databas. Datorn skyddas av lösenord. Filen skyddas av lösenord.

Delstudie 2

Undersökningsprocedur: Enbart registerstudie. Patienterna kommer inte att kontaktas.

Datainsamling och datas karaktär: Insamlade data redan tillgängliga i det svenska ryggregistret: Personnummer, typ av skolios, typ av operation. livskvalitédata (EQ5D, SRS22) samt information om längd, vikt och grad av smärta. Information kommer att kompletteras med de röntgenbilder som tagits vid klinisk rutin före och efter operation. Röntgenbilden sparas. Operationsrelaterad information (tex typ av operation, blödningsmängd) kommer att valideras genom journalgranskning.

Data från Socialstyrelsen: Från Socialstyrelsen hämtas även data från öppen- och slutenvårdsregistret för jämförelse av antal opererade, diagnoser, operationer och komplikationer rörande skolios.

Informationen förs in i en databas. Datorn skyddas av lösenord. Filen skyddas av lösenord.

Delstudie 3

Undersökningsprocedur: Personer som bedöms vid Karolinska Universitetssjukhuset för bedömning av eventuell skolios kommer att tillfrågas om att delta i undersökningen. Personer som bedöms vid Karolinska Universitetssjukhuset för andra diagnoser kommer att tillfrågas om att delta i undersökningen.

Datainsamling och datas karaktär: Personnummer, diagnos, resultat av ultraljudsundersökningen.

Informationen förs in i en databas. Datorn skyddas av lösenord. Filen skyddas av lösenord.

Delstudie 4

Undersökningsprocedur: Personer i olika åldersgrupper väljs slumpmässigt ut ur befolkningsregistret. Totalt eftersträvas 300 svar. En enkät skickas (samma enkät som används i delstudie 1).

Datainsamling och datas karaktär: Personnummer, enkätsvar.

Informationen förs in i en databas. Datorn skyddas av lösenord. Filen skyddas av lösenord.

2:5 Redogör för om insamlat biologiskt material kommer att förvaras i en biobank

(Info: p. 2:5 i Vägledning till ansökan)

Med biobank avses biologiskt material från en eller flera människor som samlas och bevaras tills vidare eller för en bestämd tid och vars ursprung kan härledas till den eller de människor från vilka materialet härrör. Redogör för var och hur prover som ska sparas förvaras, kodningsprocedurer och villkor för utlämnande av

prover. Ange huvudman för biobanken. Observera att i förekommande fall ska anmälan av biobank ske till Socialstyrelsen enligt lagen (2002:297) om biobanker i hälso- och sjukvården m.m.

-

2:6 Redovisa tillgång till nödvändiga resurser under projektets genomförande

Ange vem/vilka som har ansvaret (prefekt, verksamhetschef eller motsvarande) för forskningspersonernas säkerhet vid alla enheter/kliniker där forskningspersoner ska delta. Intyg från dessa ansvariga *ska* bifogas (se p. 9 bilaga nr 9). Av intyget ska framgå att erforderliga ekonomiska, strukturella och personella resurser finns tillgängliga för att garantera forskningspersonernas säkerhet.

Forskningspersonens säkerhet: Verksamhetschefen.

Se även bilaga.

2:7 Journalföring, registrering och hantering av data (Info: p. 2:7 i Vägledning till ansökan)

Redogör för hur undersökningsprocedurer och eventuella ingrepp journalförs. Ange hur registrering och behandling av resultaten ska gå till. Om materialet ska kodas, ange proceduren, vem som förvarar kodlistor och vem eller vilka som har tillgång till dem, var och hur länge de förvaras samt om materialet kommer att anonymiseras eller förstöras. Ange om band- och videoinspelningar används. Redogör för vilken tillgänglighet datamaterialet har och hur det förvaras samt hur erforderligt sekretesskydd erhålls.

Undersökningsprocedurer journalförs på sedvanligt sätt i digitalt journalsystem. Registrering av behandlingsdata (röntgenbilder och journalinformation) extraheras från journalsystemet. Röntgenbilder sparas.

Resultatbehandling sker genom analys i statistikprogram. Kodning av data eller anonymisering kommer inte att ske. Materialet kommer att sparas tills vidare. Band eller videoinspelningar används inte.

Datamaterialet kommer att förvaras så att enbart studieansvariga kommer att ha tillgång. Material i pappersform kommer att förvaras i låst rum. Dator skyddas av lösenord. Databaser skyddas av lösenord. Endast studieansvariga kommer att förses med lösenord. Då all studieinformation är insamlad kommer resultatberäkningar att göras på anonymiserade data. Vid resultatredovisning kommer gruppvis redovisning att ske. Enskilda personer kommer inte att kunna identifieras.

2:8 Redogör för tidigare erfarenheter (egna och/eller andras) av den använda proceduren, tekniken eller behandlingen

Särskilt angeläget är att redovisning av risker för komplikationer görs tydliga och i förekommande fall med angivande av relevanta publikationer. Vid nya behandlingar av patienter, t.ex. med läkemedel, bör anges hur många patienter (med aktuell eller annan åkomma) som tidigare erhållit föreslagen behandling, läkemedelsdosering (eller annan dosering) samt hur långa behandlingsperioder som studerats.

Delstudie 1: All teknik och alla undersökningsprocedurer är klinisk rutin. Korsettbehandling kan ge komplikation i form av lokal rodnad och skavsår men kan i allmänhet botas genom justering av korsetten.

Delstudie 2: Enbart registerstudie. Inga risker.

Delstudie 3: Ultraljudsundersökningen används av oss i en annan studie. Den ger inte upphov till skador eller smärta vid användning.

Delstudie 4: Enbart enkätstudie. Inga risker.

3. Uppgifter om forskningspersoner

3:1 Hur görs urvalet av forskningspersoner? (Info: p. 3:1 i Vägledning till ansökan)

Med forskningsperson avses en levande människa som forskningen avser. Ange urvalskriterier (inklusion och exklusion). Redogör för på vilket sätt forskaren kommer i kontakt med/får kännedom om lämpliga forskningspersoner. Ange om rekrytering sker från egna/andras tidigare eller pågående studier. Om annonsering sker, *ska* annonsmaterialet insändas som bilaga (se p. 9 bilaga nr 3). Om t.ex. barn eller personer som tillfälligt eller permanent inte är kapabla att ge ett eget informerat samtycke ska ingå i projektet, ska detta särskilt motiveras. Om vissa grupper (t.ex. kvinnor, barn eller äldre) utesluts från deltagande i projektet ska detta särskilt motiveras.

Delstudie 1: Urvalskriterier:

Skolios med Cobbvinkel 25-40 grader. Minst ett år av förväntad längdtillväxt kvar.

Ingen känd orsak till skoliosen (får inte ha tex muskeldystrofi, CP, spinal muskelatrofi eller kot- eller revbensmissbildning).

Barn (8 år eller äldre) kommer att ingå i projektet. Hos barn som är 15 år eller yngre inhämtas samtycke från vårdnadshavare.

Delstudie 2: Personer som opererats för skolios i Sverige.

Delstudie 3: Personer som bedöms på ortopedmottagningen för skolios. Personer på ortoped- eller barnortopedmottagningen som bedöms för andra orsaker än skolios.

Delstudie 4: Personer slumpvis utvalda ur befolkningsregistret.

3:2 Ange relationen mellan forskare/försöksledare och forskningspersonerna

- [x] Behandlare (t.ex. läkare, psykolog, sjukgymnast) - forskningsperson (t.ex. patient, klient)
- [ ] Kursgivare (lärare) - student
- [ ] Arbetsgivare - anställd
- [ ] Annan relation som kan tänkas medföra risk för påverkan. Beskriv:

3:3 Redogör för det statistiska underlaget för studiepopulationens (-ernas)/ undersökningsmaterialets (-ens) storlek (Info: p. 3:3 i Vägledning till ansökan)

Redovisa statistisk styrka, så kallad "power"- beräkning eller redovisa motsvarande överväganden som tydliggör studiens möjligheter att besvara frågeställningarna.

Delstudie 1: Antal: 135 personer (45 i varje grupp) vid power 80% och alfa 0,05.

Denna beräkning är baserad på följande antaganden: (1) att 15% av barnen i behandling med korsett eller postural träning kommer att få en ökad Cobbvinkel, (2) 45% av barnen i gruppen som enbart tränar kommer att få en ökad Cobbvinkel (historiska data) och (3) ett förväntat bortfall under studien på 20%.

Delstudie 2: Observationsstudie på registermaterial. Powerberäkning bedöms ej vara relevant.

Delstudie 3: Kross-sektionell observationsstudie för att skaffa information om normalvärden vid ultraljudsmätning av skelettålder hos personer med och utan skolios. Powerberäkning ej utförd.

Delstudie 4: Kross-sektionell enkätstudie för att skaffa information om normalvärden för enkäten SRS22 hos personer i befolkningen. Powerberäkning ej utförd.

3:4 Ange om forskningspersonerna kan komma att inkluderas i flera studier samtidigt eller i annan/andra studie (-er) i nära anslutning till denna? I så fall vilken typ av forskning? (Info: p. 3:4 i Vägledning till ansökan)

Ja, forskningspersonerna i delstudie 1 och 3 kan komma att inkluderas i någon eller några av de övriga studier om skolios som pågår vid ortopedklinikerna. Dessa innefattar blodprovstagning för studie av metabolism och genetik, mätning av livskvalité, benkvalitet och utseende av ryggradens krökning med olika metoder. Dessa studier är enbart observationsstudier och kommer inte ha någon inverkan på den behandling som ges i denna studie.

3:5 Vilket försäkringsskydd finns för de forskningspersoner som deltar i projektet?

Det åligger forskningshuvudmannen att kontrollera att befintliga försäkringar täcker eventuella skador som kan uppkomma.

Patientförsäkringen gäller.

3:6 Vilken ekonomisk ersättning eller andra förmåner utgår till de forskningspersoner som deltar i projektet och när betalas ersättningen ut? Utförligare beskrivning kan lämnas i bilaga.

(Info: p. 3:6 i Vägledning till ansökan)

Ersättning för obehag och besvär. Belopp (före skatt):	☐ Ja	☒ Nej
Ersättning för förlorad arbetsinkomst	☐ Ja	☒ Nej
Reseersättning	☐ Ja	☒ Nej
Befrielse från kostnader för läkemedel		
Befrielse från andra kostnader. Vilka?		
Andra förmåner. Vilka?	Trisslotter eller motsvarande ersättning kan	

komma att delas ut till deltagarna

När betalas ersättningen ut?

Ingen ersättning betalas ut ☐

4. Information och samtycke (Info: Forskningspersonsinformation)

4:1 Proceduren för och innehållet i den *information* som lämnas då forskningspersoner tillfrågas om deltagande

Beskriv hur och när information ges och vad den innehåller. Ange vem som informerar. Normalt ska en kortfattad och lättförståelig skriftlig information ges. Denna skriftliga information *ska* bifogas ansökan (se p. 9 bilaga nr 4). Om ingen eller ofullständig information ges, måste skälen för detta noggrant anges.

Delstudie 1: Patienter som uppfyller inklusionskriterierna informeras om studien av behandlande läkare, sjukgymnast eller forskningssköterska. Den tilltänkta forskningspersonen ges muntlig och skriftlig information (se bilaga). Om personen vill delta skrivs skriftligt samtycke på. Då samtycket skrivits på sker lottning. Lottning görs av person som inte är direkt involverad i vården av personen.

Delstudie 2: Registerstudie. Patienten har tidigare medgivit att delta i ryggregistret. Inget annat samtycke inhämtas.

Delstudie 3: Forskningspersoner som uppfyller inklusionskriterierna informeras om studien av behandlande läkare, sjukgymnast eller forskningssköterska. Den tilltänkta forskningspersonen ges muntlig och skriftlig information (se bilaga). Om personen vill delta skrivs skriftligt samtycke på.

Delstudie 4: Forskningspersonen informeras per brev. Skriftlig information ges. Om personen vill delta skrivs skriftligt samtycke på.

4:2 Hur och från vem inhämtas *samtycke*?

Beskriv proceduren; vem som frågar, när detta sker och hur samtycket dokumenteras. Utförlig redovisning är särskilt viktig då barn eller personer med nedsatt beslutskompetens ingår i studien, likaså vid studier av en grupp/grupper, t.ex. föreningar, organisationer, företag, kyrkosamfund, församlingar eller skolklasser.

Samtycke inhämtas från forskningspersonen. Om forskningspersonen är 15 år eller yngre inhämtas också samtycke från vårdnadshavare.

5. Forskningsetiska överväganden

5:1 Redogör för de risker som deltagandet kan medföra samt möjliga komplikationer

Dessa kan vara t.ex. fysisk skada, smärta, obehag eller integritetsintrång som projektet innebär eller kan innebära. Ange vilka åtgärder som har vidtagits för att förebygga de risker som nämns ovan samt vilken beredskap som finns för att hantera sådana komplikationer. Ange vilka/de metoder som kommer att användas för att efterforska, registrera och rapportera oönskade händelser.

Delstudie 1: Med korsettbehandlingen i sig finns en risk för hudpåverkan som tex skavsår. Vi kommer att efterfråga sådana komplikationer. Om de uppstår kommer korsetten att justeras.

5:2 Redogör för förutsebar nytta för de forskningspersoner som ingår i projektet

Delstudie 1: Forskningspersonerna får en mycket noggrann uppföljning. Det innebär att allt kommer att göras för att återbesökstider och undersökningar skall göras på rätt tid.

5:3 Gör en egen värdering av förhållandet risk - nytta för de forskningspersoner som deltar

Delstudie 1: Idag är normalbehandling för de personer som är aktuella för denna studie behandling med korsett minst 20 timmar per dygn. I denna studie kommer barnen att lottas till korsettbehandling, hållningsträning eller enbart konditionsträning. För de som ingår i gruppen "enbart träning" finns en risk. Denna är att de går miste om en effektiv behandling, förutsatt att korsettbehandling eller hållningsträningen visar sig vara effektiv. Gruppen "enbart träning" kommer att följas lika noga som de övriga. Om skoliosen skulle öka signifikant kommer dessa personer att erbjudas korsettbehandling, men med en fördröjning jämfört med om de initialt blivit lottade till behandlingsgruppen.
Sannolikt sker idag en överbehandling med korsett då inte alla personer får en ökning av sin skolios ens utan behandling. Idag har vi inte möjlighet att välja ut dessa personer. Det finns alltså ett antal personer i observationsgruppen som inte kommer att behöva behandling och därmed skulle ha direkt nytta av studien, dvs de skulle slippa använda korsett.
Vid Karolinska Universitetssjukhuset görs idag ofta röntgenundersökningar av barn med skolios 1-2 gånger per år. På de flesta andra skolioskliniker i landet sker röntgen oftast 2 gånger per år. Det innebär att patienterna i delstudie 1 kommer att röntgas något oftare än det som är klinisk

rutin vid vår klinik. Å andra sidan kommer de inte att röntgas oftare än det som är det normala vid andra kliniker i landet. Tätare röntgen kan också tänkas innebära fördelar för de som har en snabbt ökande skolios.

5:4 Identifiera och precisera om etiska problem t.ex risk - nytta i ett vidare perspektiv kan uppstå inom eller genom projektet

Här kan redovisas om exempelvis vissa grupper kan komma att utpekas/få hjälp som ett resultat av studien.

Delstudie 1: Nyttan med projektet är att vi får bättre evidens för behandling av skolios. Projektets resultat kan komma att påverka barn både med och utan skolios. Varje år screenas nämligen i Sverige cirka 200.000 barn inom skolhälsovården för att hitta de 300-500 barn som bör behandlas med korsett. Skulle det visa sig att korsettbehandling är ineffektiv kommer det att påverka policyn avseende screeningverksamhet inom skolhälsovården. Data kommer att presenteras utan att enskilda kommer att kunna identifieras.

Delstudie 2: Registerstudie. Patienterna har behandlats inom ramen för den ordinarie sjukvården. Data kommer att presenteras utan att enskilda kommer att kunna identifieras.

Delstudie 3: Undersökningen med ultraljud av skelettåldern bedöms inte innebära någon risk och heller inget obehag. Data kommer att presenteras utan att enskilda kommer att kunna identifieras.

Delstudie 4: Enkätstudien medför ingen risk. Det finns en möjlighet att de som tillfrågas om deltagande upplever detta som ett integritetsintrång. Att besvara frågorna i enkäten kan också uppfattas som ett integritetsintrång. Vi bedömer dock att detta intrång inte är stort. De personer som mot förmodan skulle uppleva ett integritetsintrång har möjlighet att avstå att besvara enkäten. Data kommer att presenteras utan att enskilda kommer att kunna identifieras.

6. Redovisning av resultaten

6:1 Hur garanteras forskningshuvudmannen och medverkande forskare tillgång till data (anges vid t.ex. uppdragsforskning) och vem ansvarar för databearbetning och rapportskrivning?

(Info: p. 6:1 i Vägledning till ansökan)

Studieansvariga kommer att ansvara för databearbetning och rapportskrivning.

6:2 Hur kommer resultaten att göras offentligt tillgängliga? Kommer studien att insändas för publicering i tidskrift eller publiceras på annat sätt?

Ange i vilken form resultaten planeras offentliggöras samt tidsplan för detta.

Ja, studieresultat kommer att publiceras på vetenskapliga möten och i vetenskapliga tidskrifter.

6:3 På vilket sätt garanteras forskningspersonernas rätt till integritet när materialet offentliggörs/publiceras?

Redovisas resultat på statistisk gruppnivå? Beskriv procedurer eller metoder för avidentifiering/anonymisering.

Ingående forskningspersoners data kommer inte att kunna identifieras vid offentliggörande. Resultat kommer att presenteras på gruppnivå.

7. Redovisning av ekonomiska förhållanden och beroendeförhållanden

Redovisning enligt punkterna 7:1-7:3 syftar till att tydliggöra alla direkta eller indirekta förhållanden, som kan tänkas påverka forskarens relation till forskningspersonerna (vid t.ex. informations-, samtyckes-, genomförandeprocedurer).

7:1 Vid uppdragsforskning

Ange uppdragsgivaren t.ex. ett företag (vid klinisk läkemedelsprövning eller prövning av andra nya produkter), en organisation eller en myndighet.

Namn:
Adress:

Kontaktperson:
Telefon/mobiltelefon:

Ange uppdragsgivarens relation till forskningshuvudmannen/medverkande forskare, t.ex. anställningsförhållande

7:2 Redovisa eventuella ekonomiska överenskommelser med uppdragsgivare eller andra finansiärer (namn, belopp)

Vid klinisk läkemedelsprövning bör hänvisning ske till ingånget avtal med sjukvårdshuvudmannen. Liknande överenskommelser kan förekomma vid annan uppdragsforskning och ska redovisas på samma sätt. Separata överenskommelser med den/de som ska genomföra forskningen ska redovisas. Belopp som kommer att erhållas för studien/ersättning till kliniken/genomföraren, vad ersättningen ska täcka och ev. belopp som erhålls per forskningsperson, ska också anges här (se p. 9 bilaga nr 12).

Inga

7:3 Redovisa forskningshuvudmannens, huvudansvarig forskares och medverkande forskares egna intressen

Här redovisas t.ex. aktieinnehav, anställning, konsultuppdrag i finansierande företag, eget företag som kan få (direkt eller indirekt) ekonomisk vinst av forskningen (se p. 9 bilaga nr 12).

Inga intressekonflikter finns.

8. Undertecknande

Behörig företrädare för sökande forskningshuvudman enligt p. 1:2

Ort: Stockholm Datum: 1201

Signatur:

Namnförtydligande: Lennart Adamsson

Tjänstetitel: Verksamhetschef

Undertecknad forskare som genomför projektet (kontaktperson) enligt p. 1:3 intygar härmed att forskningen kommer att genomföras i enlighet med ansökan

Ort: Stockholm Datum: 120120

Signatur:

Namnförtydligande: Paul Gerdhem

Tjänstetitel: Docent, överläkare

9. Förteckning över bilagor (Info: p. 9 i Vägledning till ansökan)

Dokument som, i tillämpliga fall, ska bifogas *om inte motsvarande information finns i blanketten* har markerats med x. Markera de bilagor som skickas in med denna ansökan.

Insänd med ansökan	Bil nr	Beskrivning	Klinisk läkemedels-prövning	Annan forskning
☐	1	Deltagande forskningshuvudmän och medverkande forskare (kontaktpersoner) vid forskning där mer än en forskningshuvudman deltar. Info p. 1:5	X	X
☒	2	För fackmän avsedd forskningsplan, vid behov även för lekmän avsedd bilaga. Info p. 2:1 och i Vägledning till forskningsplan/forskningsprotokoll (program)	X	X
☐	3	Annonsmaterial för rekrytering av forskningspersoner. Info p. 3:1 och i Vägledning till ansökan p. 3:1	X	X
☒	4	Skriftlig information till dem som tillfrågas. Info p. 4:1 och i Forskningspersonsinformation	X	X
☒	5	Enkät, frågeformulär. Info p. 2:4	X	X
☐	6	Gemensam EU blankett (gäller fr.o.m. den 1 maj 2004), gäller även vid ändring.	X	
☐	7	Sammanfattning av protokollet på svenska	X	
☐	8	Prövarhandbok alt. bipacksedel/produktresumé/IB	X	
☒	9	Intyg från verksamhetschef/motsv. om resurser och om forskningspersonernas säkerhet. Info p. 2:6	X	X
☒	10	CV för forskare (samma som p. 1:3) med huvudansvar för genomförandet, redovisa forskarens (-arnas) kompetens av relevans för studien. Info i Vägledning till ansökan p. 1:3	X	X
☐	11	Beskrivning av ersättning till forskningspersoner. Info p. 3:6 och i Vägledning till ansökan p. 3:6	X	X
☐	12	Överenskommelser med uppdragsgivare/finansiär om t.ex. anställningsförhållanden, bidrag/ersättning till prövningsplats, sjukvårdshuvudman, forskningshuvudman eller forskare. Info p. 7:2 och p. 7:3	X	X

Övriga bilagor som bifogas ansökan:

Purpose and aims

The purpose of this project is to fill knowledge gaps identified in the literature regarding the conservative treatment of adolescent idiopathic scoliosis (AIS). Recent systematic literature reviews on physical exercise and bracing in the treatment of AIS have provided some low quality evidence for their efficacy in preventing the progress of scoliosis and reducing surgery rates. There is however a clear lack of randomised controlled trials comparing the effectiveness of these interventions. From a health care delivery and patient related perspective, knowledge of the treatments effect in terms of cost effectiveness, quality of life, psychological wellbeing and biophysical changes is lacking. Furthermore, it is unknown which patient related, situational, or environmental characteristics moderate treatment effects and which processes mediate treatment effects. This specific knowledge could help in developing more personalised and efficient health care intervention delivery which may influence treatment outcomes more positively.

The primary aim of this research proposal is to improve evidence for the costs and effects of conservative treatments for AIS which may help prevent the progression of scoliosis and the need for surgical interventions. Secondary aims include improving knowledge of factors moderating and mediating treatment effect in terms of patients quality of life, psychological wellbeing as well biophysical factors. In addition, the quality of life of persons operated for scoliosis and persons without scoliosis will be studied and skeletal age determined by ultrasound will be evaluated.

Survey of the field

Adolescent idiopathic scoliosis is a three-dimensional structural deformation of the spine with lateral shift and rotation of the vertebrae in otherwise normal children during puberty (9). The prevalence of AIS with a curvature over 10° has been reported to be between 2-12% of the population (11,19). For curvatures between 20-40° the frequency is reduced to 0.3-0.5% while deformities over 40° make up only 0.1% of the total AIS population (15,24). The majority of patients with AIS initially do not present with symptoms but rather spinal asymmetry noted during childhood primary health care screening. These children and adolescents are often referred to specialist orthopedic clinics for assessment, longitudinal observation and treatment until skeletal maturity is reached (22). By early adulthood, the majority of patients with scoliosis suffer from back pain and if the curve progresses to be very large, even pulmonary dysfunction and psychological distress can occur (25).

Several theories propose that during the adolescent period of skeletal growth, bone deformation may occur in the event of vertebral body weakness or an imbalance of muscle forces as well as joint flexibility. A recent review of literature concerning the association of low bone mineral density and AIS reported an osteoporosis prevalence of 20-38 percent (29). Physical activity is a requirement for normal growth and development during childhood and adolescents. It is well documented that physical exercise is associated with improvements in not only muscle strength, aerobic fitness and motor development but also bone density which may help decrease the risk of osteopenic related bone deformation (5,14).

A recent report from the Swedish National Institute of Public Health (SNIPH) showed that only 45% of 11, 13 and 15 year olds are physically active 60 minutes per day, 5-7 day a week. An additional 30 percent were physically active 3-4 days a week and 25% 0-2 days a week (23). Literature even suggests that patient's with AIS treated conservatively and those treated with surgery have lower participation in sports activities than age matched controls (21). Guidelines from the SNIPH and the World Health Organisation (WHO) recommend a minimum of 60 minutes of physical activity every day. Both moderate and strenuous activity should be included and can be

divided up into several shorter sessions during the day. Activities should be enjoyable and as varied as possible to provide aerobic fitness, muscular strength, flexibility, speed, shorter reaction times and coordination (20,28). Moderate-intensity physical activity is referred to as working hard enough to raise your heart rate and break a sweat, yet still being able to carry on a conversation.

Interventions using a social cognitive theory based approach including health educational and motivational approaches to improve adolescent's habitual physical activity level have been recommended based on previous literature (5). Such interventions include changes on both a school based level and even health care based level. The Swedish government have even implemented legislation for changes in school physical education programs focused on offering students the opportunity for daily physical activity throughout the hole school day as a complement to physical education classes.

In the case of AIS, it is possible that newly diagnosed patients with reinforcement from parents may automatically restrain from physical activity in concern for it contributing to scoliosis progression. Research has however shown that systematic exercising is not associated with the development of AIS (12). Orthopedic teams involved in longitudinal screening of scoliosis progression therefore need to play a main role in educating and motivating patients with the support of parents and teachers to maintain recommended physical activity levels to minimize the possible contributing effects inactivity.

In general, conservative treatments such as physical exercise or bracing aim to prevent and possible reverse progression of scoliosis curves. Published guidelines for the conservative treatment of scoliosis recommend that conservative treatments for AIS be implemented when curve magnitudes of 25-45° are apparent (26). Surgical intervention for AIS is generally first considered when curve magnitudes reach >50° (8).

A recent systematic literature review investigating the effectiveness of physical exercise in the treatment of AIS reported the results of 1 randomised controlled trial, 9 prospective cohort controlled trials, 8 prospective observational cohort studies and 2 retrospective cohort studies (10). Together, the studies provide a limited evidence base which suggests the efficacy of physical exercise in reducing the scoliosis progression rate and/or improving Cobb angles compared to observation. It is however unclear if there is a difference in the effect of physical exercise alone or if physical exercise needs to have a postural corrective approach. Similarly, a recent literature review investigating the effectiveness of brace treatment of AIS reported the results of 2 prospective cohort controlled trials and 18 longitudinal case control studies (13). Together, the existing studies provide a limited evidence base for the efficacy of bracing preventing the progression of scoliosis without reduced quality of life.

Two randomized trials comparing brace treatment with observation are currently being conducted, one in Canada (http://www.controlled trials.com/ISRCTN81 733841/scoliosis) and one in the US (http://www.srs.org/professionals/positions/?id=62). Another trial from Holland has been terminated due to recruitment difficulties (6).

Both of the on-going randomized trials use a Boston brace like orthosis worn during day and night as the active treatment. The patients in the control arm are observed only. The Boston brace is made of hard plastic and stretches from under the arms to the pelvis. It is custom made and corrects the scoliotic curvature of the spine when worn. The psychological impact when using the brace 20-23 hours per day should not be underestimated. In one study, 27% of the brace treated patients reported that the treatment had a major negative effect on their lives (7), and our clinical impression is that this is one of the reasons to the poor compliance that is often seen. Recently, preliminary data from Gothenburg suggested that approximately 8 hours of night-time bracing with an over-corrective

brace was as effective as bracing during 23 hours per day (27).

Night-time bracing is attractive since you wear the brace a limited amount of time. The brace does not restrict activities during daytime. Our clinical impression is that the psychological concern for a teenager is much less when compared to brace treatment day and night-time, which increases the possibility of good compliance. There have been no controlled studies on night time bracing versus observation only. Several uncontrolled trials have been published, indicating an immediate corrective effect on the scoliosis by the brace (17).

Only one low quality study has compared bracing with physical exercise showing no statistical differences in the reduction or progression of scoliosis curves between the groups. To draw valid conclusions about the effectiveness of postural specific physical exercise and brace therapeutic interventions compared to a self mediated activity observational group, a randomised controlled trial research design is needed to compare the interventions.

Nationwide data on surgical treatment of scoliosis does not exist today. Quality of life in persons surgically treated for scoliosis will be investigated. Similar questionnaires will also be distributed to controls in the population.

Skeletal age is important as a predictor of scoliosis progression. Today, all acknowledged methods use radiology. Information of skeletal age measured with ultrasound will be obtained in this project to get normative data in persons with and without scoliosis. If successful, such methodology could replace the current radiological methods.

Project description

Research questions for study number 1-4

Study 1: Are there significant differences in outcomes for AIS patients receiving postural corrective physical activity or brace treatments, and what differences exist compared to a group that performs non-specific physical activity?

Study 2: What are the impacts of scoliosis on quality of life? How does quality of life change after surgical treatment?

Study 3: What is the relation between skeletal age, measured with ultrasound, and chronological age in persons with and without scoliosis?

Study 4: What is the result from questionnaires regarding scoliosis and quality of life when answered by persons without scoliosis?

Study 1: Research design

Newly referred patients to the Karolinska University Hospital orthopaedic clinic receive an orthopedic consultation including x-ray assessment of the spine. The magnitude of the curve in the frontal plane is assessed by measuring the correlating Cobb angle. Patients between 8-17 years of age with a primary curve of 25-40° with no prior treatment and skeletal immaturity assessed by the level of ossification on wrist x-rays as well as pelvic x-ray. Patients with estimated remaining growth of at least one year will be eligible for the study.

Patients will be excluded from the study if the pathogenesis of the scoliosis is not idiopathic but due to a neuromuscular, neurological, congenital malformation or trauma related comorbidity.

After assessment, patients fulfilling the inclusion and exclusion criteria will be asked to give written and signed consent for participation in an open book randomised controlled trial comparing 3 forms of health care management which are current practice in different clinics internationally. On recruitment to the study, baseline measures will be taken and random concealed block allocation using sealed opaque envelopes will be used to form the intervention groups. We estimate that all participants have been recruited in approximately 3 years. Patients will be reassessed every 6 months.

Figure 1. *Flow chart of the randomised controlled study*

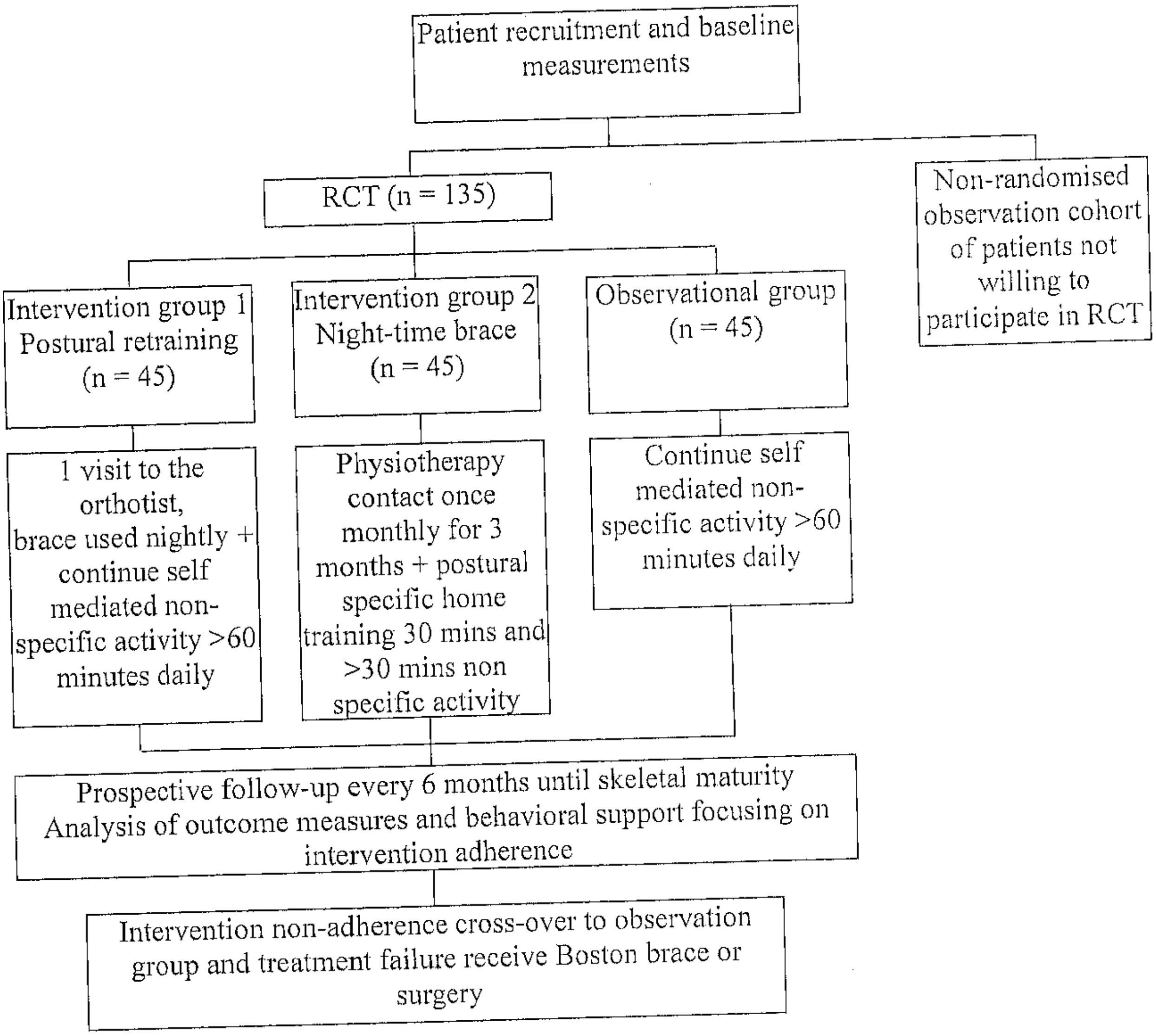

The end point failure of treatment is defined as an increase of the Cobb angle of at least 6 degrees on two consecutive x-rays, when compared to the x-ray performed at time of inclusion. Based on figures from previous literature, a failure rate of 45% in the observational self-mediated activity group and a 15% failure rate in the brace and postural training group is hypothesised. Given a significance level of 5%, a power of 80% and consideration for dropout of up to 20%, an estimated 45 patients are required in each of the intervention groups. Patients choosing not to participate in the study due to the randomisation process will be used as a comparison group. For this observational group, self mediated activity will not be encouraged, so the natural course of these patients will be followed. Patients recruited to the brace group but not adhering to the intervention protocol will cross-over to observational group recommended to continue self-mediated physical activity. Patients who have Cobb angle progress >6 degrees and ultimately reaching the end point

failure of treatment will not remain in the study but will be offered Boston brace treatment. In the event that a Cobb angle surpasses 50 degrees, patients will be offered surgical treatment.

Study 1: Interventions

1. Postural corrective physical activity:
The first phase of the intervention will be delivered in 3 x 1 hour sessions, once per month during the first 3 months. Goals at the neuromotor and biomechanical levels are directed towards postural control and spinal stability, while the goals at the bodily and psychological levels are directed towards aerobic functioning and development of a positive body image. The following steps are used:

Session 1

- Postural rehabilitation: Training awareness of body posture and postural deficits by using visual (mirror) and tactile (contact in the various postures) and verbal (therapist) feedback.
- Active self-correction on the 3 spatial planes:
  - Training of the awareness of curve apex translation towards concavity on the frontal plane. For example, in the case of a double-curve scoliosis, teaching how to execute thoracic curve horizontal translation and then lumbar curve horizontal translation.
  - Training awareness of correction on the sagittal plane to ensure thoracic kyphosis and lumbar lordosis within normal ranges. With the patient leaning with their back against a wall, the patients train pelvis anteversion and a kyphotisation movement at the thoracic level. When the patient becomes aware of the movement, it is then done without feedback from the leaning against the wall.
  - By combining the previous movements in the frontal and sagittal planes, cross-sectional derotation then occurs in the third plane.
- Training muscular stabilisation and endurance in corrective postures: Muscle endurance strengthening aims at developing paravertebral, abdominal, lower limb and scapulo-humeral girdle muscles through isometric contractions in lying, sitting and standing positions. It uses loads that are one-third to two-thirds of maximal load in active self-correction. Patients are asked to execute an active self-correction movement and to hold it for the entire duration of isometric contraction of the chosen muscles.
- Training muscular stabilisation and endurance in corrective postures during closed kinetic chain weight bearing functional movements
- Self-mediated home postural training program to be performed daily until session 2.

Session 2
Development of balance reactions: Training muscular stabilisation and endurance in corrective postures during open and closed kinetic chain functional movements on unstable planes, developed with growing difficulties. Self-mediated home postural training program to be performed daily until session 3.

Session 3
Neuromotor integration: Progressively developing the ability to apply active auto-correction through integration in everyday behaviours such as during walking, running, lifting, oculo-manual tasks and sport specific tasks. During this conclusive phase of treatment, ergonomic education is also given. The postural specific self-mediated home training program is to be performed with moderate intensity at least for 30 minutes daily, for the entirety of the study. The patients are even recommended to continue with other non-specific self-mediated physical activities previously

performed prior to the study >30 minutes daily to fulfill the general recommended quota of >60 minutes moderate intensity physical activity per day. A training diary will be implemented to follow and motivate the patient's training behaviour.

The second part of the intervention is the follow-up assessment and a cognitive behavioural therapy approach to the reinforcement of the postural training and other self mediates activities. The patients training diary will provide a basis for motivational discussion. This will be performed in conjunction with clinical reassessments every 6 months.

2. Brace treatment
An over-corrective brace will be specifically designed to the patient's individual scoliosis type and will be prescribed for night time use. A cognitive behavioral therapy approach to reinforcement of brace use will be performed in conjunction with reassessment every 6 months. Patients are encouraged to also continue with non-specific self-mediated physical activities of moderate intensity at least 60 minutes daily, for the entirety of the study. A training diary will be implemented to follow and motivate the patient's training behaviour.

3. Non-specific self-mediated physical activity
Patients are encouraged to also continue with non-specific self-mediated physical activities of moderate intensity at least 60 minutes daily, for the entirety of the study. A cognitive behavioral therapy approach to reinforcement of physical activity will be performed in conjunction with reassessment every 6 months. A training diary will be implemented to follow and motivate the patient's training behaviour.

Study 1: Measurements
The following measures will be used to assess treatment outcome and to assess possible moderation and mediation of treatment effects.

Background data: The following data will be collected at the first visit after inclusion in the study:

- Patient demographics such as age, gender, ethnicity, weight, height, health status and medications

Primary outcome measures recorded at baseline and every 6 months for the entirety of the study:

- Cobbs angle: The Cobb angle is formed by the inclination of the upper end plate of the upper end vertebra and the inclination of the lower end plate of the lower end vertebra measured on posterior to anterior view x-ray pictures. The end point failure of treatment was defined as an increase of the cobb angle of at least 6 degrees from the time of the first x-ray on two consecutive x-rays.

Secondary outcome measures recorded at baseline and every 6 months for the entirety of the study :

- SRS-22 quality of life questionnaire
- EQ5D /-Y/ quality of life questionnaire
- International Physical Activity Questionnaire (IPAQ) short form.
- Surgical rates

Treatment effect modifiers:
The following biophysical and psychosocial measures will be performed at baseline and every 6 months after the start of the intervention:

- Biophysical measures
    - 3D Surface topography picture sequence
        - Static postural indices in 3 spatial planes

        - Spinal proprioception for sense of repositioning
        - Test of trunk stiffness to the application and removal of a load during a progressive isoinertial lifting evaluation (PILE)
    - Isometric muscle endurance (Sorensen test, Shirado test)
    - Test of balance: Static, clinical balance is tested with sharpened Romberg's position with eyes closed and the non-dominant foot in front of the dominant foot. Dynamic, clinical balance is tested walking in a figure-of-eight.
    - Physical exertion test. Use of measures of Borg's RPE (rating of perceived exertion)
    - Spirometry

- Psychosocial variables
    - Mental health subscale of the SRS-22
    - SAQ – Scoliosis appearance questionnaire

At each 6 month follow-up additional questions regarding protocol fulfilment (According to CONSORT guidelines), patient and health care worker satisfaction and adverse affects will be asked:

- Adherence to the intervention protocol.
- Use of external health care services.
- Use of medicines (analgesics).
- Report of adverse events and symptoms.

Study 2

There is today international consensus that patients with progressive scoliosis with Cobb angles of more than 50 degrees are offered surgery. Life quality data of patients operated for surgery are scarce. In addition to the randomised controlled study outlined above, data from the Swedish spine register will be used to describe life quality before and after surgery for patients operated for scoliosis. We already have permission to extract data from the spine register regarding patients operated for scoliosis. At present data from about 1300 patients are available. The validity of these data regarding number of surgeries and occurrence of complications will be compared with data from the National Board of Health and Welfare (Socialstyrelsens) data for in- and outpatients surgically treated for scoliosis. Patients will be included through the primary ICD-codes for scoliosis (M41), matched with the surgical codes for corrective spine surgery or spinal fusion (NAT or NAG). In addition, the validity of the radiological information in the registry will be compared with the actual x-rays taken before and after surgery. The patients will not be contacted.

Study 3

One of the best predictors of scoliosis progression is estimating the skeletal age. When the skeleton is mature the risk of progression is low for those individuals with scoliosis with Cobb angles of less than 50 degrees. Clinically it is not always easy to determine skeletal age. The methods used today involve different radiological techniques. A new instrument (BonAge®, Sunlight Medical, Tel Aviv, Israel) using ultrasound has been developed. We are currently using this technique in another study. We now plan to explore this further by using it on a consecutive cohort of children and adolescents with scoliosis and a control group of children without scoliosis. We estimate to investigate 150 persons with scoliosis and 150 persons without scoliosis for comparison and to be able to create normative Swedish data. The method is without any risks and takes only five minutes to perform.

Study 4

Despite frequent use of specific questionnaires to estimate quality of life and back problems in patients with scoliosis, these questionnaires have until recently not been tested in persons without scoliosis, and so far only in an English speaking population. We intend to change this by distributing some of the most commonly used questionnaires to controls taken from the population. We aim to obtain answers from 500 individuals, evenly distributed in different age groups from 8-70 years of age.

Time Plan

Under the duration of 3 years it is estimated that 135 patients can be recruited to form the 3 patient groups in study 1. Data sampling for study 2 has already been performed. Data sampling for studies 3 and 4 will start during 2012. The following represents a timeline for the project (Figure 2).

Figure 2. *Research program timeline (numbers refer to study numbers mentioned above).*

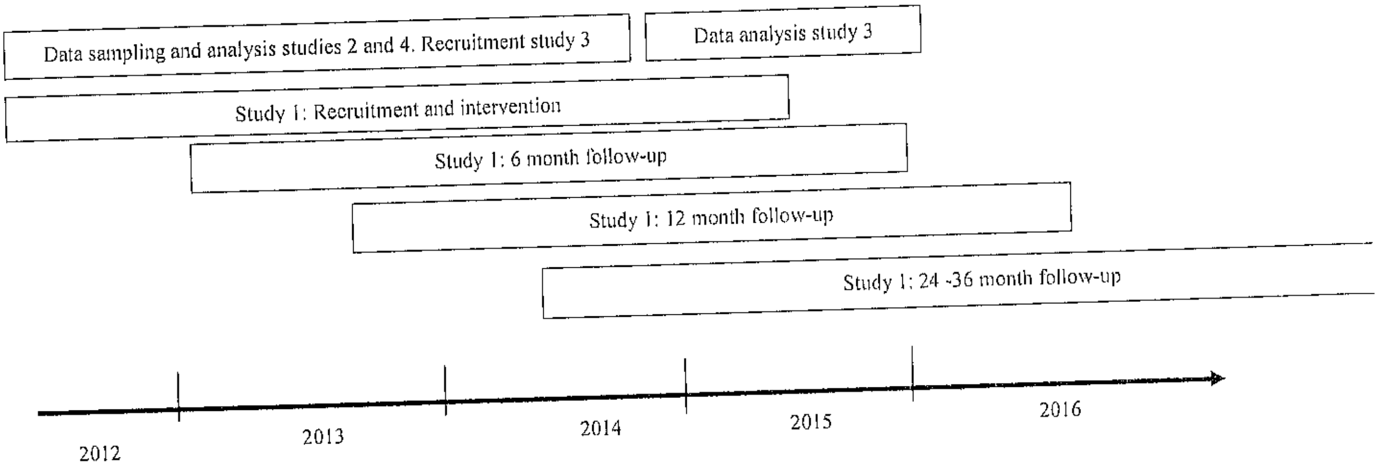

Significance

Societal and patient level
This research program has the potential to fill current knowledge gaps in the literature by providing evidence for the effect of brace treatment or physiotherapeutic intervention on scoliosis. If treatment is ineffective, the subsequent conclusion may be that the current school screening should be terminated.

Gender differences
In epidemiological studies, AIS has been shown to be more prevalent in females. There are no reports on the importance of gender on the effectiveness of conservative interventions for AIS or gender differences in factors moderating or mediating treatment. The impact of gender on the results will be specifically analysed in the publications.

Ethical Considerations
Before the start of the project, ethical approval will be applied for at the Regional Ethical Review Board in Stockholm. No medical risks or obvious issues from a patient's integrity standpoint are associated with participation in the studies.

Implementation
It is expected that the publication of research results in peer reviewed scientific journals and presentation of results at national and international meetings and congresses will help with the wide implementation of results.

The principal investigators experience

Associate Professor Paul Gerdhem has extensive experience in handling large studies for longer time periods. His expertise involves more than 10 years of clinical management of scoliosis, and ongoing research including etiological aspects of scoliosis.

Dr Allan Abbott has worked clinically with AIS patients for over 10 years. Earlier research has investigated the influence of psychological factors showing them to be strong mediators explaining up to 50% of the disability and quality of life problems observed in chronic back pain patients. We have even observed that psychological factors predict the outcome of spinal surgery and that a rehabilitation approach focused on behavioural medicine and physical activity improves outcome in these patients (1-4). We now shift focus of our research to investigate interventions to reduce the need for spinal correction surgery.

References

1. Abbott AD, Tyni-Lenné R, Hedlund R. Early rehabilitation targeting cognition, behaviour and motor function after lumbar fusion. A randomized controlled trial. *Spine* 2010;35(8):848-857.

2. Abbott AD, Tyni-Lenné R, Hedlund R. The influence of psychological factors on pre-operative levels of pain intensity, disability and HRQOL in lumbar spinal fusion surgery patients. *Physiotherapy* 2010;96(3):213-21.

3. Abbott AD, Hedlund R, Tyni-Lenné R. Patient's experience post-lumbar fusion regarding back problems, recovery and expectations in terms of the international classification of functioning, disability and health. *Disabil Rehabil* 2010; Nov 17.

4. Abbott AD, Tyni-Lenné R, Hedlund R. Leg pain and psychological variables predict outcome 2-3 years after lumber fusion surgery. *Eur Spine J* 2011;Jan.

5. Berg, U. Children and younger people, in FYSS – Physical activity in the prevention treatment of disease., Ståhle A, Editor. 2010, Profession associations for physical activity and the Swedish National Institute of Public Health: Stockholm.

6. Bunge EM, Habbema JD, de Koning HJ. A randomised controlled trial on the effectiveness of bracing patients with idiopathic scoliosis: failure to include patients and lessons to be learnt. Eur Spine J 2010;19:747-53.

7. Danielsson AJ, Wiklund I, Pehrsson K, Nachemson AL. Health-related quality of life in patients with adolescent idiopathic scoliosis: a matched follow-up at least 20 years after treatment with brace or surgery. Eur Spine J 2001;10:278-88.

8. Dolan LA, Weinstein SL. Surgical rates after observation and bracing adolescent idiopathic scoliosis. Spine 2007;32:S91-S100.

9. Hawes MC, O'Brien JP. The transformation of spinal curvature into spinal deformity: Pathological processes and implications for treatment. Scoliosis 2007;1:3.

10. Fusco C, Zaina F, Atanasio S, Romano M, Negrini A, Negrini S. Physical exercise in the treatment of adolescent idiopathic scoliosis: An updated systematic review. Physiotherapy theory and practice 2011; 27(1):80-114.

11. Grivas TB, Vasiliadis E, Mouzakis V, Mihas C, Koufopoulos G. Association between adolescent idiopathic scoliosis prevalence and age at menache in different geographic latitudes. Scoliosis 2006;1:9.

12. Kenanidis E, Potoupnis ME, Papavasiliou KA, Sayehg FE, Kapetanos GA. Adolescent idiopathic scoliosis and exercising. Is there truly a liason? Spine 2008;33:2160-2165.

13. Maruyama T, Grivas TB, Kaspiris A. Effectiveness and outcomes of brace treatment: A systematic review. Physiotherapy theory and practice 2011;27(1):26-42.

14. McKay HA, Petit MA, Schutz RW, Prior JC, Barr SI, Khan KM. Augmented trochanteric bone mineral density after modified physical education classes: a randomized school-based exercise intervention study in prepubescent and early pubescent children [see comments]. J Pediatr. 2000;136(2):156- 62.

15. Nachemson AL, Lonstein JE, Weinstein SL. Report of the prevalence and natural history committee of the scoliosis research society. Scoliosis Research Society. Denver, 1982.

16. Nachemson AL, Peterson LE. Effectiveness of treatment with a brace in girls who have adolescent idiopathic scoliosis. A prospective, controlled study based on data from the brace study of the Scoliosis Research Scociety. J Bone Joint Surg Am. 1995;77:815-22.

17. Negrini S, Minozzi S, Bettany-Saltikov J, et al. Braces for idiopathic scoliosis in adolescents. Cochrane Database Syst Rev 2010:CD006850.

18. Nicolopoulos KS, Burwell RG, Webb JK. Stature and its components in adolescent idiopathic scoliosis: cephalo-caudal in the trunk of girls. J Bone Joint Surg Br 1985: 67: 594–601.

19. Nissinen M, Heliovaara M, Ylikoski M, Poussa M. Trunk asymmetry and screening for scoliosis: A longitudinal cohort study of pubertal school children. Acta Paediatrica 1993; 82:77-82.

20. Nordic Council of Ministers. (2004). *Nordic nutrition recommendations 2004. Integrating nutrition and physical activity. NORD 2004:13*. Copenhagen: Nordic Council of Ministers.

21. Parsch D, Gärtner V, Brocai DRC, Carstens C, Schmitt H. Sports activity of patients with idiopathic scoliosis at long-term follow-up. Clinical journal of sports medicine. 2002; 12: 95-98.

22. Rigo M. Patient evaluation in idiopathic scoliosis: Radiographic assessment, trunk deformity and back asymmetry. Physiotherapy theory and practice 2011;27(1):7-25.

23. Statens folkhälsainstitut (2011) Skolbarns vanor inom fysisk aktivitet, tv-tittande och datoranvändning. R 2011 (6) Östersund: Statens Folkhälsoinstitut.

24. Weinstein SL 2001. Adolescent idiopathic scoliosis. Natural history. In: Weinstein SL (ed), The Pediatric Spine. Principles and Practice, 2nd ed, pp355-369. Philadelphia, lippincott Williams & Wilkins.

25. Weinstein SL, Dolan LA, Spratt KF et al. Health and function of patients with untreated idiopathic scoliosis: A 50 year natural history study. JAMA 2003;289: 559-67.

26. Weiss HR, Negrini S, Rigo M, Kotwicki T, Hawes MC, Grivas TB, Maruyama T, Landauer F. Indications for conservative management of scoliosis (guidelines). Scoliosis 2006; 1:5.

27. Wessberg P, Nordwall A. Night-time Providence bracing compared to full time Boston bracing in adolescent idiopathic scoliosis. A prospective randomized study (Abstract). . In: SICOT/SIROT/SOF; Aug 30-Sep 3; 2010 Aug 30-Sep 3; Gothenburg; 2010.

28. World Health Organization. (2010). *Global recommendations on physical activity for health*. Geneva: World Health Organization [WHO].

29. Xin-Feng L, Hai L, Zi-De L, Li-Yang D. Low bone mineral density in adolescent idiopathic scoliosis. Eur Spine J, 2008;17:1431-1440.

PATIENTINFORMATION TILL BARN OCH UNGDOMAR

-STUDIE AV SKOLIOSBEHANDLING -

Du har fått diagnosen idiopatisk skolios. Din läkare har bedömt att risken att du skall få en ökning av din skolios är så stor att du rekommenderas behandling. Den vanligaste behandlingsformen för skolios i Sverige och internationellt är korsett. Trots att korsettbehandling funnits i många år så har den aldrig utvärderats på bästa möjliga sätt. Det gör att vi är osäkra på hur effektiv behandlingen är. Det enda sättet att ta reda på om behandlingen är effektiv är att jämföra den med andra behandlingar. Andra lovande behandlingar för skolios är olika former av träning. Dessa är idag inte heller utvärderade på bästa sätt.

Vi vill nu fråga dig om du kan tänka dig att delta i en studie. Om du vill delta i studien innebär det att du kommer att lottas till någon av tre olika behandlingar; korsettbehandling, hållningsträning eller konditionsträning.

Korsettbehandlingen innebär att du får bära en korsett på natten. Du skall använda den minst 8 timmar per natt. Korsetten anpassas och tillverkas speciellt för dig av en ortopedingenjör. Återbesök hos läkare och sjukgymnast sker var 6:e månad. Utöver detta får du träffa ortopedingenjör vid behov.

Hållningsträningen innebär att du får träffa sjukgymnast 3 gånger under 3 månader och får ett hemträningsprogram som du sköter själv. Återbesök hos läkare och sjukgymnast sker var 6:e månad.

Konditionsträningen innebär att du får träffa sjukgymnast och får ett träningsprogram för konditionen. Återbesök hos läkare och sjukgymnast sker var 6:e månad.

Vid återbesöken var 6:e månad genomförs också en rutinmässig röntgenundersökning av din rygg och din hand. Röntgenundersökningen och stråldosen motsvarar den som de flesta barn och ungdomar med skolios får vid den normala uppföljningen av en skoliosbehandling. Du kommer att få besvara ett frågeformulär vid starten av studien och vid varje återbesök.

Deltagande i studien är frivilligt. Du kan ångra dig när som helst. Om du ångrar dig och inte längre vill vara med i undersökningen, så kommer vi att förstöra de frågeformulär du besvarat. Om din skolios skulle öka kommer du att erbjudas korsettbehandling med en annan typ av korsett, som du använder minst 20 timmar per dygn. Behandlingen kommer att pågå tills du vuxit klart. De flesta flickor har vuxit klart vid 16 års ålder och de flesta pojkar har vuxit klart vid 18 års ålder. Utöver behandlingen kommer du att erbjudas att komma på återbesök upp till 10 år efter att behandlingen avslutats.

Det är inte säkert att du själv får några fördelar genom att delta i studien. Du kommer dock att få en mer omfattande undersökning och tätare återbesök än de som inte deltar i studien. Om du inte är med i studien kommer du att erbjudas den idag vanligaste typen av korsett, som används 20-23 timmar per dygn.

Ansvarig för studien
Paul Gerdhem
Docent, överläkare
Allan Abbott
Med Dr, sjukgymnast
Ortopediska kliniken och sjukgymnastkliniken
Karolinska Universitetssjukhuset, Huddinge

141 86 Stockholm
Telefon 08-58587211, 0736-994409

Forskningshuvudman
Stockholms läns landsting

PATIENTINFORMATION TILL FÖRÄLDRAR

-STUDIE AV SKOLIOSBEHANDLING -

Ditt barn har fått diagnosen idiopatisk skolios. Den behandlande läkaren har bedömt att risken att ditt barn skall få en ökning av sin skolios är så stor att ditt barn rekommenderas behandling. Den vanligaste behandlingsformen för skolios i Sverige och internationellt är korsett. Trots att korsettbehandling funnits i många år så har den aldrig utvärderats på bästa möjliga sätt. Det gör att vi är osäkra på hur effektiv behandlingen är. Det enda sättet att ta reda på om behandlingen är effektiv är att jämföra den med andra behandlingar. Andra lovande behandlingar för skolios är olika former av träning. Dessa är idag inte heller utvärderade på bästa sätt.

Vi vill nu fråga dig om du kan tänka dig att ditt barn kan delta i studien. Deltagande i studien innebär att ditt barn kommer att lottas till någon av tre olika behandlingar; korsettbehandling, hållningsträning eller konditionsträning.

Korsettbehandlingen innebär att ditt barn får bära en korsett på natten. Korsetten skall användas minst 8 timmar per natt. Korsetten anpassas och tillverkas speciellt för ditt barn av en ortopedingenjör. Återbesök hos läkare och sjukgymnast sker var 6:e månad. Utöver detta får ni träffa ortopedingenjör vid behov.

Hållningsträningen innebär att ditt barn får träffa sjukgymnast 3 gånger under 3 månader och får ett hemträningsprogram som ni sköter själva hemma. Återbesök hos läkare och sjukgymnast sker var 6:e månad.

Konditionsträningen innebär att ditt barn får träffa sjukgymnast och får ett träningsprogram för konditionen. Återbesök hos läkare och sjukgymnast sker var 6:e månad.

Vid återbesöken var 6:e månad genomförs också en rutinmässig röntgenundersökning av din rygg och din hand. Stråldosen motsvarar [beskrivning i enlighet med strålskyddskommitténs förslag]. Du kommer att få besvara ett frågeformulär vid starten av studien och vid varje återbesök.

Deltagande i studien ~~är~~ ditt barns frivilligt. Ni kan ångra er när som helst. Om ni ångrar er och inte längre vill vara ~~med~~ i undersökningen, så kommer vi att förstöra de frågeformulär ditt barn besvarat. Om ~~din~~ skolios skulle öka kommer ditt barn att erbjudas korsettbehandling med en annan typ av korsett, som används minst 20 timmar per dygn. Behandlingen kommer att pågå tills ditt barn vuxit klart. De flesta flickor har vuxit klart vid 16 års ålder och de flesta pojkar har vuxit klart vid 18 års ålder. Utöver behandlingen kommer ditt barn att erbjudas att komma på återbesök upp till 10 år efter att behandlingen avslutats.

Det är inte säkert att ditt barn får några fördelar genom att delta i studien. Ditt barn kommer dock att få en mer omfattande undersökning och tätare återbesök än de som inte deltar i studien. Om ditt barn inte är med i studien kommer ditt barn att erbjudas korsettbehandling av annan typ, som används minst 20 timmar per dygn.

Ansvarig för studien
Paul Gerdhem
Docent, överläkare
Allan Abbott
Med Dr, sjukgymnast
Ortopediska kliniken och sjukgymnastkliniken
Karolinska Universitetssjukhuset, Huddinge

141 86 Stockholm
Telefon 08-58587211, 0736-994409

Forskningshuvudman
Stockholms läns landsting

INFORMERAT SAMTYCKE

Undertecknad har tagit del av den skriftliga informationen. Jag har förstått att mitt deltagande i studien rörande skolios är helt frivilligt och kan avbrytas när helst jag vill utan närmare förklaring. Jag ger mitt tillstånd till att journalkopior och röntgenbilder inhämtas av de studieansvariga.

Datum:____

Personnummer:____________________________________

Namn patient (texta):____________________________________

Underskrift:______________________________________

Om du är 15 år eller yngre:

Namn vårdnadshavare (texta):___________________________

Underskrift av vårdnadshavare:__________________________

Namn vårdnadshavare (texta):___________________________

Underskrift av vårdnadshavare:__________________________

Telefonnummer (mobilnummer eller hemnummer):_______________

PATIENTINFORMATION TILL BARN OCH UNGDOMAR

-STUDIE AV SKELETTÅLDER -

Skelettålder är ett sätt att bestämma skelettets mognadsgrad. Detta görs normalt med hjälp av röntgen. Vi vill nu prova en ultraljudsmetod för att bestämma skelettåldern. Ultraljud används idag till många olika saker inom sjukvården för att titta på kroppens inre organ. Ultraljud är ofarligt, gör inte ont och hörs inte.

Vi vill nu fråga dig om du kan tänka dig att delta i en studie. Om du vill vara med så använder vi ultraljud för att bestämma skelettåldern i handleden. Undersökningen tar cirka fem minuter och görs i samband med ditt besök på mottagningen.

Deltagande i studien är frivilligt. Du kan ångra dig när som helst. Om du ångrar dig och inte längre vill vara med i undersökningen, så kommer vi att ta bort den information som vi sparat från din undersökning.

Ansvarig för studien
Christos Topalis
Leg läk, forskarstuderande
Barnortopedkliniken, Astrid Lindgrens barnsjukhus
Karolinska Universitetssjukhuset, Solna
08-51770000

Paul Gerdhem
Docent, överläkare
Ortopediska kliniken
Karolinska Universitetssjukhuset, Huddinge
141 86 Stockholm
Telefon 08-58587211, 0736-994409

Forskningshuvudman
Stockholms läns landsting

PATIENTINFORMATION TILL FÖRÄLDRAR

-STUDIE AV SKELETTÅLDER -

Skelettålder är ett sätt att bestämma skelettets mognadsgrad. Detta görs normalt med hjälp av röntgen. Vi vill nu prova en ultraljudsmetod för att bestämma skelettåldern. Ultraljud används idag till många olika saker inom sjukvården för att titta på kroppens inre organ. Ultraljud är ofarligt, gör inte ont och hörs inte.

Vi vill nu fråga dig om du kan tänka dig att ditt barn deltar i en studie. Om du vill att barn skall vara med så använder vi ultraljud för att bestämma skelettåldern i handleden. Undersökningen tar cirka fem minuter och görs i samband med besöket på mottagningen.

Deltagande i studien är frivilligt. Ni kan ångra er när som helst. Om ni ångrar er och inte längre vill vara med i undersökningen, så kommer vi att ta bort den information som vi sparat från undersökningen.

Ansvarig för studien
Christos Topalis
Leg läk, forskarstuderande
Barnortopedkliniken, Astrid Lindgrens barnsjukhus
Karolinska Universitetssjukhuset, Solna
08-51770000

Paul Gerdhem
Docent, överläkare
Ortopediska kliniken
Karolinska Universitetssjukhuset, Huddinge
141 86 Stockholm
Telefon 08-58587211, 0736-994409

Forskningshuvudman
Stockholms läns landsting

INFORMERAT SAMTYCKE

Undertecknad har tagit del av den skriftliga informationen. Jag har förstått att mitt deltagande i studien rörande skelettålder är helt frivilligt och kan avbrytas när helst jag vill utan närmare förklaring. Jag ger mitt tillstånd till att journalkopior och röntgenbilder inhämtas av de studieansvariga.

Datum:____

Personnummer:____________________________________

Namn patient (texta):____________________________________

Underskrift:______________________________________

Om du är 15 år eller yngre:

Namn vårdnadshavare (texta):__________________________

Underskrift av vårdnadshavare:_________________________

Namn vårdnadshavare (texta):__________________________

Underskrift av vårdnadshavare:_________________________

Telefonnummer (mobilnummer eller hemnummer):_______________

INFORMATION OM STUDIE VID KAROLINSKA UNIVERSITETSSJUKHUSET

-STUDIE AV RYGGBESVÄR -

Hej!

Du kanske undrar varför du fått detta brev.

Vid Karolinska Universitetssjukhuset pågår flera studier om ett tillstånd som kallas skolios. Skolios är vanligt och drabbar cirka 3% av barn och ungdomar. En tiondel av dessa behöver behandling med korsett eller operation.

Vi behöver veta hälsotillståndet hos personer utan skolios för att kunna jämföra detta med de patienter vi behandlar hos oss. Vi har valt ut dig med hjälp av befolkningsregistret eftersom vi vill veta hur personer utan skolios mår. Din medverkan är viktig och kan inte ersättas av någon annan.

För att utvärdera resultaten av behandling för skolios så används flera olika frågor.

Vi skulle nu vilja ha din hjälp att besvara de bifogade frågorna. Det tar ca 15 minuter att besvara frågorna.

Deltagande i studien är frivilligt. När vi fått tillbaks det ifyllda frågeformuläret kommer vi att skicka dig en trisslott som tack för hjälpen.

Med vänlig hälsning,

Paul Gerdhem, studieansvarig
Docent, överläkare
Ortopediska kliniken och sjukgymnastkliniken
Karolinska Universitetssjukhuset, Huddinge
141 86 Stockholm
Telefon 08-58587211, 0736-994409

Forskningshuvudman
Stockholms läns landsting

INFORMERAT SAMTYCKE

Undertecknad har tagit del av den skriftliga informationen. Jag har förstått att mitt deltagande i studien rörande ryggbesvär är helt frivilligt och kan avbrytas när helst jag vill utan närmare förklaring..

Datum:____

Personnummer:____

Namn patient (texta):____

Underskrift:____

Om du är 15 år eller yngre:

Namn vårdnadshavare (texta):______________________________

Underskrift av vårdnadshavare:______________________________

Namn vårdnadshavare (texta):______________________________

Underskrift av vårdnadshavare:______________________________

Telefonnummer (mobilnummer eller hemnummer):________________

Bilaga 5

Skolios och ärftlighet

Tack för att du vill delta i projektet skolios och ärftlighet!

Enkäten består av 6 delar. Vissa frågor kan upplevas snarlika men för oss är det viktigt att få svar på alla. Om du skulle hoppa över eller missa någon fråga kan det hända att vi ringer dig för att be om en komplettering. Om du redan nu undrar något går det bra att ringa på telefon 0736-994409 måndag-fredag kl 9-16.

Då du besvarat enkäten skickar du den till oss i det bifogade portofria svarskuvertet.

Tack på förhand!

Paul Gerdhem, docent, överläkare
Allan Abbott, medicine doktor, sjukgymnast
Ortopedkliniken, K54
Karolinska Universitetssjukhuset Huddinge
141 86 Stockholm

Dagens datum ....

Namn...................................

Personnummer.........................

Gatuadress............................

Postnummer...........................

Ort......................................

Mobilnummer:........................

E-postadress .... Jag saknar e-postadress ☐

Del 1 Allmänna frågor

KÖN. Man ☐ Kvinna ☐

VIKT. Aktuell vikt: .... kg

LÄNGD. Aktuell längd: .... cm

SKO0. Har du skolios: Ja☐ Nej ☐

Om du har skolios:

SKO1. Ungefär vid vilken ålder fick du diagnosen skolios ? .... års ålder Minns ej ☐

SKO2. Har du fått någon behandling? Ingen ☐ Korsett ☐ Operation ☐ Annan ☐

Om du markerat "annan behandling" ovan:

SKO3. Vilken behandling fick du i så fall? ....

Om du markerat "korsett" ovan:

KORS1. Vid vilken ålder började du din korsettbehandling? .... års ålder

KORS2. Har du avslutat korsettbehandlingen? Ja ☐ Nej ☐

Om du avslutat korsettbehandlingen:

KORS4. Vid vilken ålder avslutade du din korsettbehandling? .... års ålder

KORS5. Vi vill att du uppskattar det genomsnittliga antal timmar per dygn som du använde/använder korsetten:

0-4 timmar per dygn ☐ *1.*
4-8 timmar per dygn ☐ *2.*
8-12 timmar per dygn ☐ *3.*
12-16 timmar per dygn ☐ *4.*
16-20 timmar per dygn ☐ *5.*
20-24 timmar per dygn ☐ *6.*

Om du opererats för skolios:

OP1. Vid vilken ålder opererades du?...........................år

Livsstil | Ja | Nej

	Ja	Nej
RÖK. Röker Du?	☐	☐
MAT1. Äter/dricker du mjölk eller mjölkprodukter ?	☐	☐
MAT2. Är du vegetarian eller vegan?	☐	☐
SKOLA. Går Du i skola eller studerar?	☐	☐
ARBETE1. Förvärvsarbetar Du?	☐	☐

Om du arbetar:

ARBETE2. **Om** Du arbetar, vad arbetar Du med? ....

ARBETE3. Vilken karaktär har ditt arbete (kryssa i en ruta)?

1. ☐ Mest stillasittande arbete
2. ☐ Lätt arbete men även rörligt till viss del. Går en del men bär eller lyfter vanligtvis inte tyngre saker
3. ☐ Måttligt tungt arbete. Går mycket och lyfter dessutom ganska mycket
4. ☐ Tungt kroppsarbete

MOTION1. Sysslar du med motions- eller tävlingsidrott? Ja ☐ Nej ☐

Om du svarat ja ovan:

MOTION1A. I så fall, sedan vilken ålder? Från ...... års ålder

MOTION2. Har du sysslat med motions- eller tävlingsidrott tidigare? Ja ☐ Nej ☐

MOTION2A+B. I så fall, i vilken ålder? Från ...... års ålder, till ... ... års ålder

RYGG1. Har Du några ryggbesvär? Ja ☐ Nej ☐

Om du svarat ja ovan:

RYGG2. I så fall, hur ofta?

Dagligen ☐ Någon gång i veckan ☐ Någon gång i månaden ☐

RYGG3. Om Du har ryggbesvär, inskränker de Dina aktiviteter på något sätt? Ja☐ Nej☐

RYGG4. Har Du några nackbesvär? Ja☐ Nej☐

Om du svarat ja ovan:

RYGG4A. I så fall, hur ofta?

Dagligen ☐ Någon gång i veckan ☐ Någon gång i månaden ☐

RYGG5. Om Du har nackbesvär, inskränker de Dina aktiviteter på något sätt? Ja☐ Nej☐

Sjukdomar

SJD1. Har du någon kronisk sjukdom? Ja ☐ Nej ☐

SJD2. Om du har någon kronisk sjukdom, besvara även nedanstående frågor:

1. ☐ Hjärtsjukdom – vilken?...
2. ☐ Neurologisk sjukdom- vad för någon?..........................
3. ☐ Diabetes/Sockersjuka
4. ☐ Astma
5. ☐ Annan lungsjukdom- vad för någon?...........................
6. ☐ Epilepsi
7. ☐ Annan kronisk sjukdom- vad för någon?........................

ALL1. Har du några allergier? Ja ☐ Nej ☐

Läkemedel

LÄK1. Tar du några mediciner/läkemedel? Ja ☐ Nej ☐

Om du tar läkemedel, besvara även nedanstående frågor:

I så fall, vilken/vilka?

LÄK13. Läkemedlets namn .... Tagit sedan (årtal) ....

LÄK14. Läkemedlets namn .... Tagit sedan (årtal) ....

LÄK15. Läkemedlets namn .... Tagit sedan (årtal) ....

LÄK16. Läkemedlets namn .... Tagit sedan (årtal) ....

LÄK17. Läkemedlets namn .... Tagit sedan (årtal) ....

LÄK18. Läkemedlets namn .... Tagit sedan (årtal) ....

Frakturer

FX1. Har Du någonsin ådragit Dig någon spricka i skelettet eller ett benbrott? Ja ☐ Nej ☐

Om du svarat ja ovan:

I så fall, i vilket ben?	Ja	Ålder	Sjukhus
FX2. Handledsbrott	☐	.......år	....
FX3. Underarmsbrott	☐	.......år	....
FX4. Överarmsbrott	☐	.......år	....
FX5. Lårbensbrott	☐	.......år	....
FX6. Annat brott	☐	.......år	....

FX7. Annat brott- vad för något?..................... ......år ....

Följande fyra frågor besvaras enbart av kvinnor:

PP. Använder Du p-piller? Ja ☐ Nej ☐

MR1. Har Du menstruationer? Ja ☐ Nej ☐

MR2. **Om** Du har eller har haft menstruationer, vid vilken ålder började de?....................

MR3. **Om** Du är lite äldre, vid vilken ålder slutade dina menstruationer? ....

Del 2 Ärftlighet

Arvet har förmodligen betydelse för den vanligaste typen av skolios. Det land och den landsdel man kommer ifrån har också stor betydelse för hur arvsanlagen ser ut. Vi skulle därför vilja veta var du och dina föräldrar är födda:

Du själv

FÖDD1. Är du född i Sverige? Ja ☐ Nej ☐

FÖDD1A. Om du är född i Sverige, i vilket län är du född (ringa in)?

Blekinge län
Dalarnas län
Gotlands län
Gävleborgs län
Hallands län
Jämtlands län
Jönköpings län
Kalmar län
Kronobergs län
Norrbottens län
Skåne län
Stockholms län
Sörmlands län
Uppsala län
Värmlands län
Västerbotten
Västernorrlands län
Västmanlands län
Västra Götalands län
Örebro län
Östergötlands län

FÖDD2. Om du inte är född i Sverige, i vilket land är du då född? ....

Mamma

FÖDDM1. Är din mamma född i Sverige? Ja ☐ Nej ☐

FÖDDM1A. Om din mamma är född i Sverige, i vilket län är hon född (ringa in)?

Blekinge län
Dalarnas län
Gotlands län
Gävleborgs län
Hallands län
Jämtlands län
Jönköpings län
Kalmar län
Kronobergs län
Norrbottens län
Skåne län
Stockholms län
Sörmlands län
Uppsala län
Värmlands län
Västerbotten
Västernorrlands län
Västmanlands län
Västra Götalands län
Örebro län
Östergötlands län
Vet inte

FÖDDM2. Om din mamma inte är född i Sverige, i vilket land är din mamma då född?

....

Pappa

FÖDDP1. Är din pappa född i Sverige? Ja ☐ Nej ☐

FÖDDP1A. Om din pappa är född i Sverige, i vilket län är han född (ringa in)?

Blekinge län
Dalarnas län
Gotlands län
Gävleborgs län
Hallands län
Jämtlands län
Jönköpings län
Kalmar län
Kronobergs län
Norrbottens län
Skåne län
Stockholms län
Sörmlands län
Uppsala län
Värmlands län
Västerbotten
Västernorrlands län
Västmanlands län
Västra Götalands län
Örebro län
Östergötlands län
Vet inte

FÖDDP2. Om din pappa inte är född i Sverige, i vilket land är han då född?

....

Föräldrar och mor/farföräldrar

ARVM1. Har din **mamma** skolios? Ja ☐ Nej ☐ Vet ej ☐
ARVM2. Om din mamma har skolios, har din mamma fått behandling?
Ja☐ Nej ☐ Vet ej☐
ARVM3. Om du svarat ja ovan, vilken behandling fick din mamma?
Korsett ☐ Operation ☐ Annan ☐ Vet ej ☐

ARVP1. Har din **pappa** skolios? Ja ☐ Nej ☐ Vet ej ☐
ARVP2. Om din pappa har skolios, har din pappa fått behandling?
Ja☐ Nej ☐ Vet ej☐
ARVP3. Om du svarat ja ovan, vilken behandling fick din pappa?
Korsett ☐ Operation ☐ Annan ☐ Vet ej ☐

ARVMM1. Har din **mormor** skolios? Ja ☐ Nej ☐ Vet ej ☐
ARVMM2. Om din mormor har skolios, har din mormor fått behandling?
Ja☐ Nej ☐ Vet ej☐
ARVMM3. Om du svarat ja ovan, vilken behandling fick din mormor?
Korsett ☐ Operation ☐ Annan ☐ Vet ej ☐

ARVMF1. Har din **morfar** skolios? Ja ☐ Nej ☐ Vet ej ☐
ARVMF2. Om din morfar har skolios, har din morfar fått behandling?
Ja☐ Nej ☐ Vet ej☐
ARVMF3. Om du svarat ja ovan, vilken behandling fick din morfar?
Korsett ☐ Operation ☐ Annan ☐ Vet ej ☐

ARVFM1. Har din **farmor** skolios? Ja ☐ Nej ☐ Vet ej ☐
ARVFM2. Om din farmor har skolios, har din farmor fått behandling?
Ja☐ Nej ☐ Vet ej☐
ARVFM3. Om du svarat ja ovan, vilken behandling fick din farmor?
Korsett ☐ Operation ☐ Annan ☐ Vet ej ☐

ARVFF1. Har din **farfar** skolios? Ja ☐ Nej ☐ Vet ej ☐
ARVFF2. Om din farfar har skolios, har din farfar fått behandling?
Ja☐ Nej ☐ Vet ej☐
ARVFF3. Om du svarat ja ovan, vilken behandling fick din farfar?
Korsett ☐ Operation ☐ Annan ☐ Vet ej ☐

Bror 1 *BRO1A.* Ålder idag? ......år *BRO1B.* Helsyskon☐ Halvsyskon ☐

BRO1SK. Har denna bror skolios? Ja ☐ Nej ☐ Vet ej ☐

BRO1BEHA. Om din bror har skolios, har din bror fått behandling?

Ja ☐ Nej ☐ Vet ej ☐

BRO2BEHB. Om du svarat ja ovan, vilken behandling fick din bror?

Korsett ☐ Operation ☐ Annan ☐ Vet ej ☐

Bror 2 *BRO2A.* Ålder idag? ......år *BRO2B.* Helsyskon☐ Halvsyskon ☐

BRO2SK. Har denna bror skolios? Ja ☐ Nej ☐ Vet ej ☐

BRO2BEHA. Om din bror har skolios, har din bror fått behandling?

Ja ☐ Nej ☐ Vet ej ☐

BRO2BEHB. Om du svarat ja ovan, vilken behandling fick din bror?

Korsett ☐ Operation ☐ Annan ☐ Vet ej ☐

Bror 3 *BRO3A.* Ålder idag? ......år *BRO3B.* Helsyskon☐ Halvsyskon ☐

BRO3SK. Har denna bror skolios? Ja ☐ Nej ☐ Vet ej ☐

BRO3BEHA. Om din bror har skolios, har din bror fått behandling?

Ja ☐ Nej ☐ Vet ej ☐

BRO3BEHB. Om du svarat ja ovan, vilken behandling fick din bror?

Korsett ☐ Operation ☐ Annan ☐ Vet ej ☐

Bror 4 *BRO4A.* Ålder idag? ......år *BRO4B.* Helsyskon☐ Halvsyskon ☐

BRO4SK. Har denna bror skolios? Ja ☐ Nej ☐ Vet ej ☐

BRO4BEHA. Om din bror har skolios, har din bror fått behandling?

Ja ☐ Nej ☐ Vet ej ☐

BRO4BEHB. Om du svarat ja ovan, vilken behandling fick din bror?

Korsett ☐ Operation ☐ Annan ☐ Vet ej ☐

Barn

BARN1. **Om** du är lite äldre: Har Du några barn? Ja☐ Nej☐

Om du inte har barn kan du fortsätta till nästa del i formuläret.

BARN2. Hur många söner? ……stycken

BARN3. Hur många döttrar? ……stycken

Son 1 *SON1A*. Ålder idag? ……år *SON1B*. Har denna son skolios? Ja☐ Nej☐

SON1BEHA. Om din son har skolios, har din son fått behandling?

Ja☐ Nej ☐ Vet ej☐

SON1BEHB. Om du svarat ja ovan, vilken behandling fick din son?

Korsett ☐ Operation ☐ Annan ☐ Vet ej ☐

Son 2 *SON2A*. Ålder idag? ……år *SON2B*. Har denna son skolios? Ja☐ Nej☐

SON2BEHA. Om din son har skolios, har din son fått behandling?

Ja☐ Nej ☐ Vet ej☐

SON2BEHB. Om du svarat ja ovan, vilken behandling fick din son?

Korsett ☐ Operation ☐ Annan ☐ Vet ej ☐

Son 3 *SON3A*. Ålder idag? ……år *SON3B*. Har denna son skolios? Ja☐ Nej☐

SON3BEHA. Om din son har skolios, har din son fått behandling?

Ja☐ Nej ☐ Vet ej☐

SON3BEHB. Om du svarat ja ovan, vilken behandling fick din son?

Korsett ☐ Operation ☐ Annan ☐ Vet ej ☐

Son 4 *SON4A*. Ålder idag? ……år *SON4B*. Har denna son skolios? Ja☐ Nej☐

SON4BEHA. Om din son har skolios, har din son fått behandling?

Ja☐ Nej ☐ Vet ej☐

SON4BEHB. Om du svarat ja ovan, vilken behandling fick din son?

Korsett ☐ Operation ☐ Annan ☐ Vet ej ☐

Dotter 1 *DOT1A.* Ålder idag? ……år *DOT1B.* Har denna dotter skolios? Ja☐ Nej☐

DOT1BEHA. Om din dotter har skolios, har din dotter fått behandling?

Ja☐ Nej ☐ Vet ej☐

DOT1BEHB. Om du svarat ja ovan, vilken behandling fick din dotter?

Korsett ☐ Operation ☐ Annan ☐ Vet ej ☐

Dotter 2 *DOT2A.* Ålder idag? ……år *DOT2B.* Har denna dotter skolios? Ja☐ Nej☐

DOT2BEHA. Om din dotter har skolios, har din dotter fått behandling?

Ja☐ Nej ☐ Vet ej☐

DOT2BEHB. Om du svarat ja ovan, vilken behandling fick din dotter?

Korsett ☐ Operation ☐ Annan ☐ Vet ej ☐

Dotter 3 *DOT3A.* Ålder idag? ……år *DOT3B.* Har denna dotter skolios? Ja☐ Nej☐

DOT3BEHA. Om din dotter har skolios, har din dotter fått behandling?

Ja☐ Nej ☐ Vet ej☐

DOT3BEHB. Om du svarat ja ovan, vilken behandling fick din dotter?

Korsett ☐ Operation ☐ Annan ☐ Vet ej ☐

Dotter 4 *DOT4A.* Ålder idag? ……år *DOT4B.* Har denna dotter skolios? Ja☐ Nej☐

DOT4BEHA. Om din dotter har skolios, har din dotter fått behandling?

Ja☐ Nej ☐ Vet ej☐

DOT4BEHB. Om du svarat ja ovan, vilken behandling fick din dotter?

Korsett ☐ Operation ☐ Annan ☐ Vet ej ☐

Andra släktingar

SLÄ1. Har någon annan släkting skolios? Ja ☐ Nej ☐ Vet ej ☐

Om du svarat ja ovan, fyll i vilka släktingar som har skolios nedan:

Släkting 1. *SLÄA.* Släktrelation till dig (moster, morbror, faster, kusin osv)?…………………

SLÄABEHA. Om denna släkting har skolios, har din släkting fått behandling?

Ja ☐ Nej ☐ Vet ej☐

SLÄABEHB. Om du svarat ja ovan, vilken behandling fick din släkting?

Korsett ☐ Operation ☐ Annan ☐ Vet ej ☐

Släkting 2. *SLÄB.* Släktrelation till dig (moster, morbror, faster, kusin osv)?....................

SLÄBBEHA. Om din släkting har skolios, har din släkting fått behandling?
Ja ☐ Nej ☐ Vet ej ☐

SLÄBBEHB. Om du svarat ja ovan, vilken behandling fick din släkting?
Korsett ☐ Operation ☐ Annan ☐ Vet ej ☐

Släkting 3. *SLÄC.* Släktrelation till dig (moster, morbror, faster, kusin osv)?....................

SLÄCBEHA. Om din släkting har skolios, har din släkting fått behandling?
Ja ☐ Nej ☐ Vet ej ☐

SLÄCBEHB. Om du svarat ja ovan, vilken behandling fick din släkting?
Korsett ☐ Operation ☐ Annan ☐ Vet ej ☐

Släkting 4. *SLÄD.* Släktrelation till dig (moster, morbror, faster, kusin osv)?....................

SLÄDBEHA. Om din släkting har skolios, har din släkting fått behandling?
Ja ☐ Nej ☐ Vet ej ☐

SLÄDBEHB. Om du svarat ja ovan, vilken behandling fick din släkting?
Korsett ☐ Operation ☐ Annan ☐ Vet ej ☐

Släkting 5. *SLÄE.* Släktrelation till dig (moster, morbror, faster, kusin osv)?....................

SLÄEBEHA. Om din släkting har skolios, har din släkting fått behandling?
Ja ☐ Nej ☐ Vet ej ☐

SLÄEBEHB. Om du svarat ja ovan, vilken behandling fick din släkting?
Korsett ☐ Operation ☐ Annan ☐ Vet ej ☐

Släkting 6. *SLÄF.* Släktrelation till dig (moster, morbror, faster, kusin osv)?....................

SLÄFBEHA. Om din släkting har skolios, har din släkting fått behandling?
Ja ☐ Nej ☐ Vet ej ☐

SLÄFBEHB. Om du svarat ja ovan, vilken behandling fick din släkting?
Korsett ☐ Operation ☐ Annan ☐ Vet ej ☐

DEL 3 Ditt allmänna hälsotillstånd (livskvalitéformulär EQ5D)

Nu kommer frågor som rör ditt allmänna hälsotillstånd. Dvs frågorna rör hela din livssituation och inte bara dina ryggrelaterade besvär. Markera, genom att kryssa i en ruta i varje nedanstående grupp vilket påstående som bäst beskriver Ditt hälsotillstånd i dag.

Rörlighet

☐ Jag går utan svårigheter
☐ Jag kan gå men med viss svårighet
☐ Jag är sängliggande

Hygien

☐ Jag behöver ingen hjälp med min dagliga hygien, mat eller påklädning
☐ Jag har vissa problem att tvätta eller klä mig själv
☐ Jag kan inte tvätta eller klä mig själv

Huvudsakliga aktiviteter *(t ex arbete, studier, hushållssysslor, familje- och fritidsaktiviteter)*

☐ Jag klarar av mina huvudsakliga aktiviteter
☐ Jag har vissa problem med att klara av mina huvudsakliga aktiviteter
☐ Jag klarar inte av mina huvudsakliga aktiviteter

Smärtor/besvär

☐ Jag har varken smärtor eller besvär
☐ Jag har måttliga smärtor eller besvär
☐ Jag har svåra smärtor eller besvär

Oro/nedstämdhet

☐ Jag är inte orolig eller nedstämd
☐ Jag är orolig eller nedstämd i viss utsträckning
☐ Jag är i högsta grad orolig eller nedstämd

Till hjälp för att avgöra hur bra eller dåligt ett hälsotillstånd är, finns den termometer-liknande skalan till höger. På denna har Ditt bästa tänkbara hälsotillstånd markerats med 100 och Ditt sämsta tänkbara hälsotillstånd med 0.

Vi vill att Du på denna skala markerar hur bra eller dåligt Ditt hälsotillstånd är, som Du själv bedömer det. Gör detta genom att dra en linje från nedanstående ruta till den punkt på skalan som markerar hur bra eller dåligt Ditt nuvarande hälsotillstånd är.

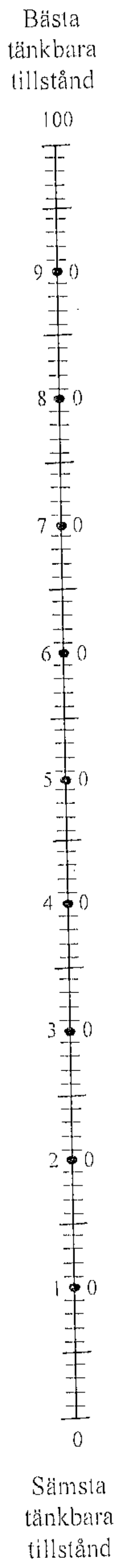

Ditt nuvarande hälsotillstånd

EQ-5D-Y

Hur är din hälsa IDAG?

Sätt ett kryss i den ruta som bäst beskriver din hälsa IDAG

Kunna röra sig

Jag har inte svårt att gå ☐

Jag har lite svårt att gå ☐

Jag har mycket svårt att gå ☐

Ta hand om mig själv

Jag har inte svårt att tvätta mig eller klä på mig själv ☐

Jag har lite svårt att tvätta mig eller klä på mig själv ☐

Jag har mycket svårt att tvätta mig eller klä på mig själv ☐

Göra vanliga aktiviteter (*till exempel gå i skolan, sport-och fritidsaktiviteter, lek, göra saker med familj eller kompisar*)

Jag har inte svårt att göra mina vanliga aktiviteter ☐

Jag har lite svårt att göra mina vanliga aktiviteter ☐

Jag har mycket svårt att göra mina vanliga aktiviteter ☐

Ha ont eller ha besvär

Jag har inte ont eller några besvär ☐

Jag har lite ont eller lite besvär ☐

Jag har mycket ont eller mycket besvär ☐

Känna sig orolig, ledsen eller olycklig

Jag är inte orolig, ledsen eller olycklig ☐

Jag är lite orolig, ledsen eller olycklig ☐

Jag är mycket orolig, ledsen eller olycklig ☐

© 2008 EuroQol Group. EQ-5D™ is a trade mark of the EuroQol Group

Hur bra är din hälsa IDAG?

Den bästa hälsa du kan tänka dig

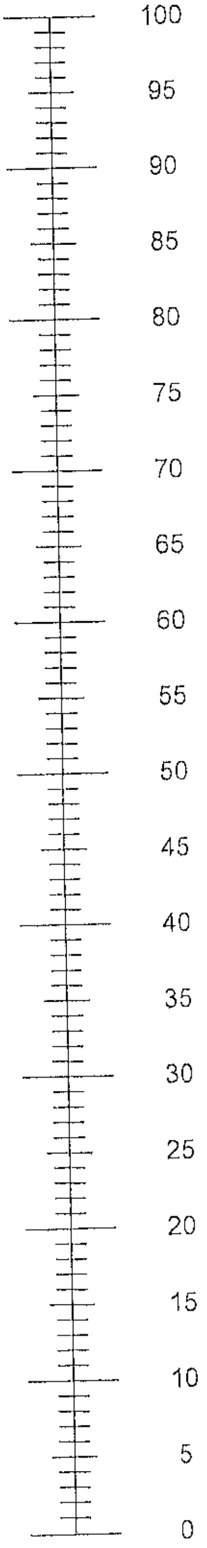

Den sämsta hälsa du kan tänka dig

- Vi vill veta hur bra eller dålig din hälsa är IDAG.
- Den här linjen går från 0 till 100.
- 100 är den bästa hälsa du kan tänka dig.
  0 är den sämsta hälsa du kan tänka dig.
- Sätt ett X på linjen som visar hur bra eller dålig din hälsa är IDAG.

© 2008 EuroQol Group. EQ-5D™ is a trade mark of the EuroQol Group

DEL 4. Utseende (Spinal appearance questionnaire)

Markera det alternativ som passar ditt utseende bäst. Kryssa enbart i ett alternativ per fråga.

1. Kroppskurvatur

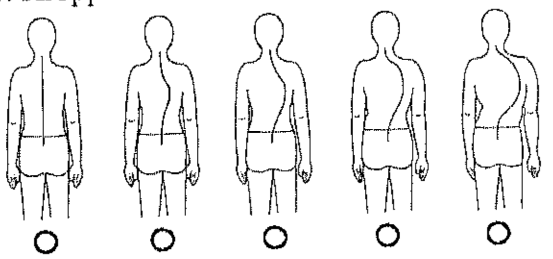

2. Revbensprominens (”puckel” i bröstryggen)

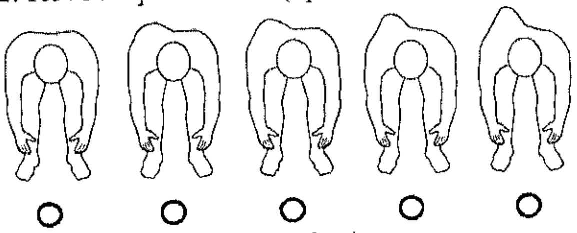

3. Flankprominens (”puckel” i ländryggen)

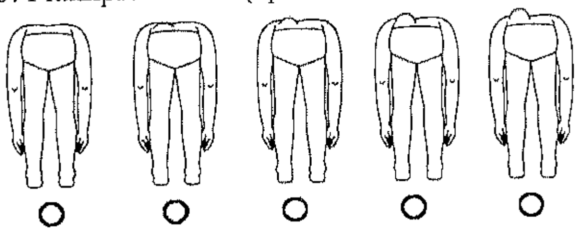

4. Huvud och bröstkorg i förhållande till bäcken

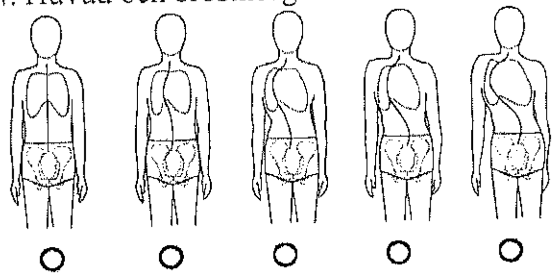

5. Huvudets position i förhållande till bäckenet

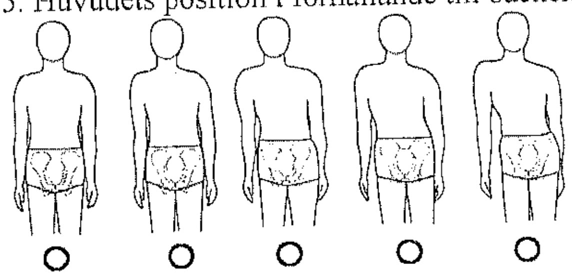

6. Axelnivå (skuldernivå)

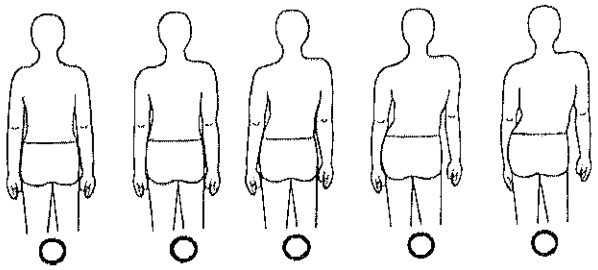

7. Ryggprominens ("puckel")

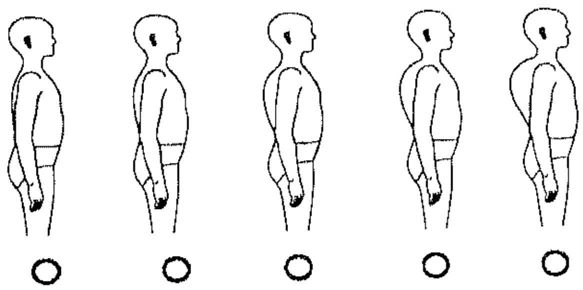

8. Vilken kategori besvärar dig mest?
(om utseendet inte besvärar dig går du vidare till nästa fråga)

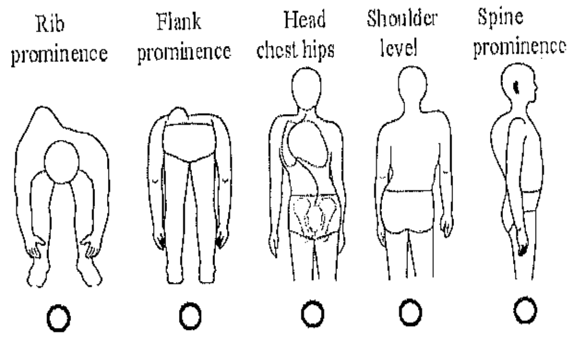

DEL 5 Ryggrelaterade besvär (formulär SRS 22r)
Vi undersöker noggrant ditt ryggtillstånd och det är viktigt att du besvarar varje fråga själv.
Ringa in det enda svar som passar bäst på varje fråga.

1. Vilket av följande beskriver bäst hur mycket smärta du har upplevt under de senaste 6 månaderna.

- [ ] Ingen
- [ ] Mild
- [ ] Måttlig
- [ ] Måttlig till svår
- [ ] Svår

2. Vilket av följande beskriver bäst hur mycket smärta du har upplevt under den senaste månaden.

- [ ] Ingen
- [ ] Mild
- [ ] Måttlig
- [ ] Måttlig till svår
- [ ] Svår

3. Under de senaste 6 månaderna, har du varit mycket nervös?

- [ ] Aldrig
- [ ] Nästan aldrig
- [ ] Ibland
- [ ] Nästan alltid
- [ ] Hela tiden

4. Hur skulle du känna dig om du var tvungen att ha din nuvarande ryggform resten av livet?

- [ ] Mycket nöjd
- [ ] Ganska nöjd
- [ ] Varken nöjd eller missnöjd
- [ ] Ganska missnöjd
- [ ] Mycket missnöjd

5. Vilken är din nuvarande aktivitetsnivå?

- [ ] Sängliggande
- [ ] Nästan inga aktiviteter
- [ ] Lätt arbete och lättare motion
- [ ] Ganska mycket arbete och ganska mycket motion
- [ ] Full aktivitet utan begränsningar

6. Hur ser du ut med kläder på?

- [ ] Mycket bra
- [ ] Bra
- [ ] Ganska bra
- [ ] Illa
- [ ] Mycket illa

7. Under de senaste 6 månaderna, har du känt dig så nere att inget har kunnat muntra upp dig?

- [ ] Mycket ofta
- [ ] Ofta
- [ ] Ibland
- [ ] Sällan
- [ ] Aldrig

8. Har du ryggsmärtor när du vilar?

- [ ] Mycket ofta
- [ ] Ofta
- [ ] Ibland
- [ ] Sällan
- [ ] Aldrig

9. Vilken är din nuvarande aktivitetsnivå på arbetet/ i skolan?

- [ ] 100% normal
- [ ] 75% normal
- [ ] 50% normal
- [ ] 25% normal
- [ ] 0% normal

10. Vilket av följande beskriver bäst utseendet på din bål, det vill säga kroppen utom huvud, armar och ben.

- [ ] Mycket bra
- [ ] Bra
- [ ] Ganska bra
- [ ] Dåligt
- [ ] Mycket dåligt

11. Vilket av följande påståenden beskriver bäst de mediciner du tar för **ryggen**?

- [ ] Inga
- [ ] Receptfria mediciner 1 gång/vecka eller mindre (t.ex. Alvedon, Voltaren, Ipren)
- [ ] Receptfria mediciner dagligen
- [ ] Receptbelagda mediciner 1 gång/vecka eller mindre (t.ex. Doloxene, Citodon, Ketogan)
- [ ] Receptbelagda mediciner dagligen

	namn	användning (ex. < 1 gång /vecka, dagligen)
Annan medicin	....	....
Annan medicin	....	....

12. Hindrar din rygg dig från att göra saker i hemmet?

- [ ] Aldrig
- [ ] Sällan
- [ ] Ibland
- [ ] Ofta
- [ ] Mycket ofta

13. Har du känt dig lugn och harmonisk de senaste 6 månaderna?

- [ ] Hela tiden
- [ ] Mesta tiden
- [ ] Ibland
- [ ] Sällan
- [ ] Aldrig

14. Känner du att ditt ryggtillstånd påverkar dina personliga relationer?

- [ ] Inte alls
- [ ] Nästan inte alls
- [ ] Lite
- [ ] Ganska mycket
- [ ] Mycket

15. Upplever du och/eller din familj ekonomiska svårigheter på grund av din rygg?

- [ ] Mycket
- [ ] Ganska mycket
- [ ] Lite
- [ ] Nästan inga
- [ ] Inga

16. Under de senaste 6 månaderna, har du känt dig nedstämd och deppig?

- ☐ Aldrig
- ☐ Sällan
- ☐ Ibland
- ☐ Ofta
- ☐ Mycket ofta

17. Under de senaste 3 månaderna, har du varit borta från arbetet eller avstått från hushållsarbete/skolan på grund av ryggont, och i så fall hur många dagar?

- ☐ 0 dar
- ☐ 1 dar
- ☐ 2 dar
- ☐ 3 dar
- ☐ 4 dar eller fler

18. Begränsar ditt ryggtillstånd dina möjligheter att gå ut med vänner/familj?

- ☐ Aldrig
- ☐ Sällan
- ☐ Ibland
- ☐ Ofta
- ☐ Mycket ofta

19. Känner du dig attraktiv med ditt nuvarande ryggtillstånd?

- ☐ Ja, mycket
- ☐ Ja, ganska
- ☐ Varken attraktiv eller oattraktiv
- ☐ Nej, inte särskilt
- ☐ Nej, inte alls

20. Har du varit lycklig under de senaste 6 månaderna?

- ☐ Ingen del av tiden
- ☐ En liten del av tiden
- ☐ En viss del av tiden
- ☐ Större delen av tiden
- ☐ Hela tiden

Fråga 21 och 22 nedan besvaras bara om du fått behandling för skolios.

21. Är du nöjd med resultatet av din ryggbehandling?

- ☐ Mycket nöjd
- ☐ Nöjd
- ☐ Varken nöjd eller missnöjd
- ☐ Missnöjd
- ☐ Mycket missnöjd

22. Skulle du välja samma behandling igen om du fick samma åkomma?

- ☐ Ja, absolut
- ☐ Ja, förmodligen
- ☐ Vet inte
- ☐ Nej, förmodligen inte
- ☐ Nej, absolut inte

DEL 6 Fysisk aktivitet (International physical activity questionnaire)

Följande frågor handlar om fysisk aktivitet. Vi är intresserade av att ta reda på all typ av fysisk aktivitet som utförs. Frågorna innefattar tid som du varit fysiskt aktiv de **senaste 7 dagarna**. Svara på frågorna även om du inte anser dig vara en aktiv person. Inkludera alla aktiviteter under såväl arbete, transporter, hushållsarbete, trädgårdsarbete, fritidsaktiviteter som planerad träning.

1. Tänk nu på alla de **mycket ansträngande** aktiviteter du utförde under de **senaste 7 dagarna. Mycket ansträngande** fysisk aktivitet innefattar aktiviteter som upplevs som mycket arbetssamma och får dig att andas mycket kraftigare än normalt. Tänk *enbart* på de aktiviteter som du utfört under minst 10 minuter i sträck.

1a. Under de **senaste 7 dagarna**, hur många av dessa dagar har du utfört arbete som är **mycket ansträngande** såsom tunga lyft, tyngre bygg- och trädgårdsarbete, aerobics, löpning eller cykling i högre tempo?

______ **dagar** ☐ Ingen sådan aktivitet ➡ Hoppa över fråga 1b

1b. Hur mycket tid tillbringade du, i genomsnitt under en sådan dag, på **mycket ansträngande** fysisk aktivitiet?

______ **minuter** ☐ Vet ej

2. Tänk nu på alla de **måttligt ansträngande** aktiviteter du utförde under de **senaste 7 dagarna. Måttligt ansträngande** fysisk aktivitet innefattar aktiviteter som upplevs som arbetsamma och får dig att andas något kraftigare än normalt. Tänk enbart på de aktiviteter som du utfört under minst 10 minuter i sträck.

2a. Under de **senaste 7 dagarna**, hur många av dessa dagar har du utfört arbete som är **måttligt ansträngande** såsom cykling, simning, måttligt bygg- och trädgårdsarbete eller annat i måttligt tempo? Inkludera ej promenader.

______ **dagar** ☐ Ingen sådan aktivitet ➡ Hoppa över fråga 2b

2b. Hur mycket tid tillbringade du, i genomsnitt under en sådan dag, på **måttligt ansträngande** aktivitet?

______ **timmar**

______ **minuter** ☐ Vet ej

3. Tänk nu på all tid du **promenerat** under de **senaste 7 dagarna.** Detta inkluderar promenader på arbetet, under transporter och under fritiden.

3a. Under de **senaste 7 dagarna**, hur många dagar har du **promenerat** i minst 10 minuter i sträck?

______ **dagar** ☐ Inga promenader ➜ Hoppa över fråga 3b

3b. Hur mycket tid per dag tillbringade du, i genomsnitt en sådan dag, på **promenader**?

______ **timmar**

______ **minuter** ☐ Vet ej

4. Tänk nu på den tid som du tillbringat **sittande** under en typisk dag, de **senaste 7 dagarna**, i samband med arbete, studier, transporter, i hemmet och på din fritid. Exempelvis tid vid skrivbordet, hemma hos vänner eller i TV-soffan.

Under de senaste 7 dagarna, **hur mycket tid har du tillbringat** sittande **under** en sådan dag?

______ timmar per dag

______ minuter per dag ☐ **Vet ej**

5. Hur mycket har du rört dig och ansträngt dig kroppsligt på fritiden under de **senaste 12 månaderna?** *Om din aktivitet varierar mellan t.ex. sommar och vinter, så försök att ta ett genomsnitt. Obs! Kryssa endast i ett alternativ!*

☐ **Stillasittande fritid**
Du ägnar dig mest åt läsning, TV, bio eller annan stillasittande sysselsättning på fritiden. Du promenerar, cyklar eller rör dig på annat sätt mindre än 2 timmar i veckan.

☐ **Måttlig motion på fritiden**
Du promenerar, cyklar eller rör dig på annat sätt under minst 2 timmar i veckan oftast utan att svettas. I detta inräknas också promenad eller cykling till och från arbetet, övriga promenader, ordinärt trädgårdsarbete, fiske, bordtennis, bowling.

☐ **Måttlig, regelbunden motion på fritiden**
Du motionerar regelbundet 1-2 gånger per vecka minst 30 minuter per gång med löpning, simning, tennis, badminton, tyngre trädgårdarbete eller annan aktivitet som gör att du svettas.

☐ **Regelbunden motion och träning**
Du ägnar dig åt t.ex. löpning, simning, tennis, badminton, motionsgymnastik, tyngre trädgårdsarbete eller liknande vid i genomsnitt minst 3 tillfällen per vecka. Vardera tillfället varar minst 30 minuter per gång.

Tack för Din medverkan!

Om du vill kommentera eller skriva ned något speciellt kan du göra det här:

....

....

....

....

Bilaga 9. Intyg verksamhetschefer

Stockholm ....

Härmed intygas att erforderliga ekonomiska, strukturella och personella resurser finns tillgängliga för att garantera forskningspersonernas säkerhet i ovanstående studie.

Lennart Adamsson, verksamhetschef
Ortopedkliniken, K54
Karolinska Universitetssjukhuset
141 86 Stockholm

KAROLINSKA
Universitetssjukhuset

Akutdivisionen
Sjukgymnastikkliniken

Etikprövningsnämnden i Stockholm
FE 289
Karolinska Institutet
171 77 Stockholm

INTYG

Härmed intygas att erforderliga ekonomiska, strukturella och personella resurser finns tillgängliga för att garantera forskningspersonernas säkerhet i studien, Skolios – resultat av behandling.

Stockholm 2012-01-09

Åsa Dedering
Verksamhetschef

Astrid Lindgrens
Barnsjukhus
vid Karolinska
Universitetssjukhuset

2012-01-03

Verksamheten för rörelseorganens sjuk-
domar och hemsjukvård (SABH), Solna
Bengt Eriksson, verksamhetschef
bengt.m.eriksson@karolinska.se

Intyg att bilägga Ansökan om etikprövning

Angående studien ”Livskvalitet och biologiska markörer” (Dnr 2009/696 31/2, delstudie 3) med Paul Gerdhem som huvudansvarig forskare och Christos Topalis som medansvarig forskare.

Härmed intygas att jag som verksamhetschef vid Verksamheten för rörelseorganens sjukdomar och hemsjukvård (SABH) har ansvaret för patienternas säkerhet under deras vård samt att erforderliga personella, strukturella och ekonomiska resurser som faller inom vår verksamhetsram finns för projektets genomförande.

Med vänlig hälsning

Bengt Eriksson
Verksamhetschef

Bil 10

CV Paul Gerdhem

Grundexamen, högskoleexamen, år, ämnesområde

1993, läkarexamen, leg läkare, 1995

Specialistkompetens, år, specialitet

2000, ortopedi

Doktorsexamen

2004, Lunds Universitet, "Risk factors for falls and fractures in elderly women", Huvudhandledare Karl Obrant, bihandledare Kristina Åkesson

Docentkompetens

Docent 2005, Lunds Universitet, Ortopedi

Nuvarande anställning

Överläkare vid ryggsektionen, Karolinska Universitetssjukhuset sedan 2006 och tills vidare, 50% forskning via klinisk forskartjänst tom 2012-12-31. Adjungerad universitetslektor Karolinska Institutet.

Handledarskap, pågående och avslutade

Pågående huvudhandledarskap för Anna Grauers (sedan 2008), Peter Elkan (sedan 2009, reg som forskarstuderande 2004), Christos Topalis (sedan 2010), Axel Wihlborg (sedan 2011). Halvtidskontroll genomförd 2011 för Peter Elkan.

Bihandledare för Robolge Lenora, Lunds Universitet, disputerad 2009.

Övrigt

Övriga anställningar

Stf studierektor på Insitutionen för klinisk vetenskap, intervention och teknik, Karolinska Institutet sedan 2008 (ca 5% av en heltid).

Förtroendeuppdrag

Ledamot av sakkunniggruppen för bedömning av tjänstetillsättning vid Karolinska Universitetssjukhuset sedan 2007.

Ledamot av disputationskommittén vid Karolinska Institutet sedan 2010.

Vice ordförande i Svensk Ryggkirurgisk Förening sedan 2010.

Vetenskaplig sekreterare i Svensk Ryggkirurgisk Förening 2006-2008.

Utmärkelser

Young investigator Award vid American Society of Bone Mineral Research, Minneapolis, USA, 2003 (försteförfattare av abstractbidrag)

John Sevastik Award vid International Research Society of Spinal Deformities i Montreal, Kanada, 2010 (seniorförfattare av abstractbidrag).

Publikationslista Paul Gerdhem

Originalarbeten

1	Ringsberg K, **Gerdhem P**, Johansson J, Obrant KJ: Is there a relationship between balance, gait performance and muscular strength in 75-year-old women? Age Ageing 1999;28:289-293.
2	**Gerdhem P**, Abdon P, Odenbring S: Hemicallotasis for medial gonarthrosis: a short-term follow-up of 21 patients. Arch Orthop Trauma Surg 2002;122:134-138.
3	**Gerdhem P**, Magnusson H, Karlsson MK, Akesson K: Ultrasound of the phalanges is not related to a previous fracture. A comparison between ultrasound of the phalanges, calcaneus, and DXA of the spine and hip in 75-year-old women. J Clin Densitom 2002;5:159-166.
4	**Gerdhem P**, Obrant KJ: Effects of cigarette-smoking on bone mass as assessed by dual-energy X-ray absorptiometry and ultrasound. Osteoporos Int 2002;13:932-936.
5	**Gerdhem P**, Ringsberg KA, Magnusson H, Obrant KJ, Akesson K: Bone mass cannot be predicted by estimations of frailty in elderly ambulatory women. Gerontology 2003;49:168-172.
6	**Gerdhem P**, Akesson K, Obrant KJ: Effect of previous and present physical activity on bone mass in elderly women. Osteoporos Int 2003;14:208-212.
7	**Gerdhem P**, Ringsberg KA, Akesson K, Obrant KJ: Influence of muscle strength, physical activity and weight on bone mass in a population-based sample of 1004 elderly women. Osteoporos Int 2003;9:768-772.
8	Ivaska KK, Hellman J, Likojarvi J, Kakonen SM, **Gerdhem P**, Akesson K, Obrant KJ, Pettersson K, Vaananen HK: Identification of novel proteolytic forms of osteocalcin in human urine. Biochem Biophys Res Commun 2003;306:973-980.
9	**Gerdhem P**, Ringsberg KAM, Åkesson K, Obrant KJ: Just one look and fracture and death can be predicted in elderly ambulant women. Gerontology 2004;5:309-314.
10	**Gerdhem P**, Mallmin H, Akesson K, Obrant KJ: Seasonal variation in bone density in postmenopausal women. J Clin Densitom 2004;7:93-100.
11	**Gerdhem P**, Brandstrom H, Stiger F, Obrant K, Melhus H, Ljunggren O, Kindmark A, Akesson K: Association of the collagen type 1 (COL1A 1) Sp1 binding site polymorphism to femoral neck bone mineral density and wrist fracture in 1044 elderly Swedish women. Calcif Tissue Int 2004;74:264-269.
12	Brandstrom H, **Gerdhem P**, Stiger F, Obrant KJ, Melhus H, Ljunggren O, Kindmark A, Akesson K: Single nucleotide polymorphisms in the human gene for osteoprotegerin are not related to bone mineral density or fracture in elderly women. Calcif Tissue Int 2004;74:18-24.
13	Nordstrom A, **Gerdhem P**, Brandstrom H, Stiger F, Lerner UH, Lorentzon M, Obrant K, Nordstrom P, Akesson K: Interleukin-6 promoter polymorphism is associated with bone quality assessed by calcaneus ultrasound and previous fractures in a cohort of 75-year-old women. Osteoporos Int 2004;10:820-826.

14	**Gerdhem P**, Ivaska KK, Alatalo SL, Halleen JM, Hellman J, Isaksson A, Pettersson K, Vaananen HK, Akesson K, Obrant KJ: Biochemical markers of bone metabolism and prediction of fracture in elderly women. J Bone Miner Res 2004;19:386-393.
15	**Gerdhem P**, Obrant KJ: Bone mineral density at old age- the influence of age at menarche and menopause. J Bone Miner Metab.2004;4:372-375.
16	**Gerdhem P**, Ringsberg KAM, Åkesson K, Obrant KJ: Clinical history and biological age predicts falls better than objective functional tests. J Clin Epidemiol 2005;28:226-232
17	Ivaska KK, Kääkönen SM, **Gerdhem P**, Obrant KJ, Pettersson K, Väänänen HK: Urinary osteocalcin as a marker of bone metabolism. Clin Chem. 2005 Mar;51(3):618-28. Epub 2005 Jan 13.
18	**Gerdhem P**, Isakssson I, Åkesson K, Obrant KJ: Increased bone density and decreased bone turnover, but no evident alteration of fracture susceptibility in elderly women with Diabetes Mellitus. Osteoporos Int. 2005 Dec 16(12):1506-1512.
19	**Gerdhem P**, Ringsberg KAM, Obrant KJ, Åkesson K: Association between 25-hydroxy vitamin D levels, physical activity, muscle strength and fractures in the prospective population-based OPRA study of elderly women. Osteoporos Int. 2005 Nov;16(11):1425-31. Epub 2005 Mar 3.
20	Obrant KJ, Ivaska KK, **Gerdhem P**, Pettersson K, Väänänen HK. Influence on biochemical markers of bone turnover of retrospectively sustained fractures. Bone. 2005 May;36(5):786-92. Epub 2005 Mar 31.
21	**Gerdhem P**, Ringsberg KAM, Åkesson K: The relation between previous fractures and balance at old age in women. Arch Phys Med Rehabil 2006 Jul;87(7):914-917
22	**Gerdhem P**, Ivaska KK, Isaksson A, Pettersson K, Vänäänen HK, Obrant KJ, Åkesson K. Associations between homocysteine, bone turnover, bone density and mortality, but not with fracture risk, in elderly women. Journal of Bone and Mineral Research. 2007 Jan (22)1:127-134, Epub 2006 Oct 9
23	Grundberg E, Åkesson K, Kindmark A, **Gerdhem P**, Holmberg A, Mellström D, Ljunggren Ö, Orwoll E, Mallmin H, Ohlsson C, Brändström H. The impact of estradiol on bone mineral density is modulated by the specific estrogen receptor-alfa cofactor insertion/deletion polymorphism. J Clin Endocrinol Metab. 2007, Mar 13; Epub ahead of print]
24	Lenora J, Ivaska KK, Obrant KJ, **Gerdhem P**. Prediction of bone loss using biochemical markers of bone turnover. Osteoporos Int. 2007 Apr 18; [Epub ahead of print]
25	Ivaska KK, **Gerdhem P**, Akesson K, Garnero P, Obrant KJ. The effect of fracture on bone turnover markers: a longitudinal study comparing marker levels before and after injury in 113 elderly women. J Bone Miner Res. 2007 Aug;22(8):1155-64.
26	Gerdhem P, Dencker M, Ringsberg K, Akesson K. Accelerometer-measured daily physical activity among octogenerians: results and associations to other indices of physical performance and bone density. Eur J Appl Physiol. 2008 Jan;102(2):173-80. Epub 2007 Sep 29.

27	**Gerdhem P**, Åkesson K. Rates of fracture in participants and non-participants in the Osteoporosis Prospective Risk Assessment study. J Bone Joint Surg Br. 2007 Dec;89(12):1627-31.
28	McGuigan FE, Larzenius E, Callreus M, **Gerdhem P**, Luthman H, Åkesson K. Variation in the BMP2 gene: Bone Mineral Density and Ultrasound in Young Adult and Elderly Women. Calcif Tissue Int. 2007 Oct;81(4):254-62. Epub 2007 Aug 29.
29	Tenne M, McGuigan F, Jansson, L, **Gerdhem** P, Obrant KJ, Luthman H, Akesson K. Genetic variation in the PTH pathway and bone phenotypes in elderly women: Evaluation of PTH, PTHLH, PTHR1 and PTHR2 genes. Bone. 2007 Dec 23; Apr 42(4):719-27. Epub 2007 Dec 23.
30	McGuigan F, Larzenius E, Callreus M, **Gerdhem P**, Luthman H, Akesson K.Variation in the bone morphogenetic protein-2 gene: effects on fat and lean body mass in young and elderly women. Eur J Endocrinol. 2008 May;158(5):661-8.
31	Ivaska KK, Lenora R, **Gerdhem P**, Akesson K, Vaananen HK, Obrant KJ. Serial assessment of serum bone metabolism markers identifies women with the highest rate of bone loss and osteoporosis risk. J Clin Endocrinol Metab. 2008, Jul 93(7): 2622-32. Epub 2008, May 6.
32	Lenora R, **Gerdhem P**, Obrant KJ, Ivaska, KK. Bone turnover markers are correlated with quantitative ultrasound of the calcaneus: 5-year longitudinal data. Osteopor Int 2009 Jul 20(7): 1225-32. Epub 2008 Oct 23.
33	Ivaska KK, **Gerdhem P**, Väänänen HK, Åkesson K, Obrant KJ. Bone turnover markers and prediction of fracture: a prospective follow-up study of 1040 elderly women for a mean of nine years. J Bone Miner Res. E-pub 2009, Dec 6.
34	McGuigan F, Kumar J, Ivaska KK, Obrant KJ, **Gerdhem P**, Akesson K. Osteocalcin gene polymorphisms influence concentration of serum osteocalcin and enhance fracture identification. J Bone Miner Res. 2010 Jun(6):1392-9. Epub 2010, Jan 29
35	Tenne M, McGuigan FE, Ahlborg H, **Gerdhem P**, Akesson K. Variation in the PTH gene, hip fracture, and femoral neck geometry in elderly women. Calcif Tissue Int. 2010(5):359-66. Epub 2010 Mar 27.
36	Swanberg M, McGuigan F, Ivaska KK, **Gerdhem P**, Lerner UH, Bucala R, Kuchel G, Kenny A, Akesson K. Polymorphisms in the macrophage migration inhibitory factor gene and bone loss in postmenopausal women. Bone. 2010(2):424-9. Epub 2010 May 12.
37	Lenora J, Akesson K, **Gerdhem P.** Effect of Precision on Longitudinal Follow-Up of Bone Mineral Density Measurements in Elderly Women and Men. J Clin Densit. 2010 Oct-Dec;13(4):407-12 [Epub ahead of print, Jun 3]
37	Lenora J, Akesson K, **Gerdhem P.** Effect of Precision on Longitudinal Follow-Up of Bone Mineral Density Measurements in Elderly Women and Men. J Clin Densit. 2010 Oct-Dec;13(4):407-12 [Epub ahead of print, Jun 3]
38	Kumar J, Swanberg M, McGuigan F, Callreus M, **Gerdhem P**, Akesson P. LRP association to bone properties and fracture and interaction with genes in the Wnt- and BMP signaling pathways. Bone 2011, Sep;49(3):343-8. [Epub ahead of print, May 27]

39	Grauers A, Rahman I, **Gerdhem P**. Heritability of Scoliosis. Eur Spine J, 2011 [Epub ahead of print, Nov 18].

Översiktsartiklar / bokkapitel

1	**Gerdhem P**. [Risk factors for falls and fracture in elderly women] (in Swedish). Incitament 2004: 6: 485-488
2	**Gerdhem P**. Falls- epidemiological aspects and prevention. In "The Year in Osteoporosis II" (2006), Ed: Woolf AD, Åkesson K
3	Karlsson, MK, Hasserius R, **Gerdhem P**, Obrant KJ, Ohlin A. [Treatments for osteoporotic vertebral compression fractures- Explosive interest for vertebro- and kyphoplasty] (in Swedish) Läkartidningen; 2005 May 23-29;102(21):1644-1646;1648
4	Karlsson MK, **Gerdhem P**, Ahlborg HG. The prevention of osteoporotic fractures. J Bone Joint Surg Br. 2005 Oct;87(10):1320-7.
5	Karlsson M, Hasserius R, **Gerdhem P**, Obrant K, Ohlin A. Vertebroplasty and kyphoplasty - New treatment strategies for osteoporotic vertebral fractures. Acta Orthop Scand 2005; 76(5): 620-627.
6	**Gerdhem P**. Vitamin D –ett ständigt aktuellt hormon viktigt för muskulär och skelettal hälsa. Medicinsk Access, 2006, April; 4-5
7	Lenora R, Ivaska KK, **Gerdhem P**. Use of bone turnover markers in osteoporosis. Clinical reviews in bone and mineral metabolism.2009, May 12, 1-14

PROTOKOLL 2012/4:2
2012-02-22

Sammanträde i Stockholm

Avdelning 4

Ordförande
Per Olding

Ledamöter med vetenskaplig kompetens
Sigurd Vitols (*klinisk farmakologi*) ***vetenskaplig sekreterare***
Erik Sundström *(geriatrik)*, deltar inte i ärende 2012/160
Tommy Linné (*barnmedicin)*
Elisabet Lidbrink (*onkologi)*, deltar inte i ärendena 2012/172, 2012/174, 2012/181 och 2012/191
Lucie Laflamme (*skadeepidemiologi, internationell hälsa)*
Guro Gafvelin (*molekylär immunologi)*
Erling Löfsjögård Nilsson (*klinisk fysiologi)*
Erik Näslund (*kirurgi)*
Lene Lindberg (*psykologi)*, deltar inte i ärende 2012/91
André Stark (*ortopedi)*

Ledamöter som företräder allmänna intressen
Rut Andersson
Roland Håkansson
Gunnar Skoglund
Åke Reisnert

Administrativ sekreterare
Eja Fridsta

§ 1 Ordföranden förklarar sammanträdet öppnat.

§ 2 Den administrativa sekreteraren anmäler att den vetenskaplige sekreteraren sedan föregående möte den 25 januari 2012 fattat beslut i 27 ärenden som avser ändring av godkännande.

§ 3 Ansökningar om etisk granskning av forskningsprojekt, se **Bilaga**.

§ 4 Ordföranden förklarar sammanträdet avslutat och meddelar att nästa sammanträde äger rum den 21 mars 2012.

Per Olding
Ordförande

Sigurd Vitols, protokollförare
Vetenskaplig sekreterare

Adress FE 289 171 77 Stockholm | **Besöksadress** Nobels väg 9 Solna | **Telefon** 08-524 800 00 | **Fax** 08-524 866 99 | **E-post** kansli@stockholm.epn.se | **Hemsida** www.epn.se

Regionala etikprövningsnämnden i
Stockholm
Sammanträde 2012-02-22

------------------------------------ Utdrag ur protokoll nr 2012/4:2 ------------------------------------

Dnr 2012/172-31/4
Föredragande:
André Stark

Sökande: Stockholms läns landsting
Behörig företrädare: Lennart Adamsson
Projekt: Skolios – resultat av behandling
Forskare som genomför projektet: Paul Gerdhem

BESLUT
Nämnden godkänner forskningen på följande villkor.

1. Information om personuppgiftslagen (PUL) ska lämnas i samtliga forskningspersonsinformationer. Se nämndens hemsida www.epn.se *snabblänkar.*
2. Rättelse av syftningsfel (näst sista stycket i informationen till föräldrar).

Hur man överklagar, se särskild information.

Beslut expedierat till behörig företrädare,
forskare och i förekommande fall till sponsor

Att utdraget överensstämmer med originalet intygar:

Lena Creutzer Waldersten
Administrativ sekreterare, beslut expedierat 2012-03-06

Stockholm Regional Ethical Review Board

Protocol 2012/4:2
22-02-2012
Meeting in Stockholm

Department 4

Chairman
Per Olding

Members with scientific competency
Sigurd Vitols *(clinical pharmacology)* ***scientific secretary***
Erik Sundström *(geriatrics)*, has not participated in case 2012/160
Tommy Linné *(pediatric medicine)*
Elisabet Lidbrink *(oncology)*, has not participated in 2012/172, 2012/174, 2012/181 and 2012/191
Lucie Laflamme *(injury epidemiology, international health)*
Guro Gafvelin *(molecular immunology)*
Erling Löfsjögård Nilsson *(clinical physiology)*
Erik Näslund *(surgery)*
Lene Lindberg *(psychology)*, has not participated in case 2012/91
André Stark *(orthopaedics)*

Lay representatives
Rut Andersson
Roland Håkansson
Gunnar Skoglund
Åke Reisnert

Administrative secretary
Eja Fridsta

§1 The Chairman opened the meeting

§2 The administrative secretary reported that the scientific secretary since the last meeting on the 25th of January 2012 has approved 27 cases relating to changes in approved ethics reviewed projects.

§3 Applications for ethical review of research projects, se appendix.

§4 The chairman closed the meeting and confirmed that the next meeting will occur on 21 march 2012.

Per Olding
Chairman

Sigurd Vitols, Minutes
Scientific secretary

Regional Ethical Review Board
in Stockholm
Meeting 22-02-2012

-----------------------------------Extract form the protocol nr 2012/4:2 --

Dnr 2012/172-31/4
Appointment
André Stark

Applicant: Stockholm country Council
Authorised representative: Lennart Adamsson
Project: Scoliosis – result of treatment
Primary Researcher: Paul Gerdhem

DECISION
The committee approves the research on the following conditions.

1. Information about the Privacy Act should be included in the explanatory statement to participants. See the committee's homepage www.epn.se *quicklinks.*
2. Correct the grammatical error (Second last paragraph I the information to parents)

How to appeal, see specific information.

Decision expedited to the authorised representative, researcher and in appropriate cases, to sponsors

---

This extract is consistent with origin certificates

Lena Creutzer Waldersten
Administrative secretary, decision expedited 06-03-2012

This is to certify that this is a true copy of the english translated version of the original swedish document

Allan Abbott
ALLAN ABBOTT
Karolinska University Hospital
0046 733816914 .... Date 12/7/2013

Address	Visiting Address	Telephone	Fax	E-post	Hemsida
FE289<br>171 77 Stockholm<br>Sweden	Nobels street 9<br>Solna	08-524 800 00	08-524 866 99	kansli@stockholm.epn.se	www.epn.se

15-04-08

Regionala etikprövningsnämnden i Stockholm
FE 289
171 77 STOCKHOLM

2015/1007-32

Studien"Skolios- resultat av behandling" godkändes av etikprövningsnämnden 2012-02-22 (Dnr 2012/172-31/4).

Jag önskar att etikprövningsnämnden tar ställning till följande ändring av projektet.

Då inklusionstakten i det som benämns delstudie 1 i den tidigare ansökan varit långsammare än beräknat (fn har ca 1/3:del av patienterna inkluderats) önskar vi utvidga studien till fler centra. Protokollet är oförändrat. Antalet patienter som skall inkluderas är oförändrat (135 st). Karolinska är fortsatt huvudansvarig för studien, utför randomisering och är fortsatt involverad i all behandling och uppföljning av patienterna. Bifogat är intyg från följande kliniker:

Ryggkliniken, Linköpings Universitetssjukhus (lokalt ansvarig: Allan Abbott)
Ortopedkliniken, Länssjukhuset Ryhov, Jönköping (lokalt ansvarig: Anna Aspberg Ahl)
Ortopedkliniken, lasarettet Västerås (lokalt ansvarig: Björn Dahlman)
Ortopedkliniken, Norrlands Universitetssjukhus, Umeå (lokalt ansvarig: Pawel Grabowski)
Ortopedkliniken, Sundsvalls sjukhus (lokalt ansvarig: Anna Grauers)
Ortopedkliniken, Mälarsjukhuset Eskilstuna (lokalt ansvarig: Ingrid Ekenman/Ylva Bodén)

Avgiften på 2000 kr har betalts in på bankgiro 794-5496, märkt med " Dnr 2012/172-31/4 Gerdhem". Kvitto på betalning bifogas.

Med vänlig hälsning,

Paul Gerdhem
Ortopedkliniken, K54
Karolinska Universitetssjukhuset, Huddinge
141 86 Stockholm
0736-994409
paul.gerdhem@karolinska.se

Regionala etikprövnings-
nämnden i Stockholm
Inkom: 2015 -06- 09
Dnr:

Bilagor:
Intyg från berörda verksamhetschefer.

Kopia för kännedom: Karl-Åke Jansson, verksamhetschef, ortopedkliniken, Karolinska Universitetssjukhuset

GODKÄNNS Dat. 150610
Sigurd Vitols
Vetenskaplig sekreterare
Regionala etikprövningsnämnden
i Stockholm

15-04-08

The Regional Ethical Review Board in Stockholm

FE289 2015/1007-32

171 77 Stockholm

The study "Scoliosis - results of treatment" was approved by the Ethical Review Board 2012-02-22 (Dnr 2012/172-31/4).

We would like the Ethical Review Board to approve the following additions to the project.

As the rate of inclusion in what is referred to as sub-study 1 in the previous application has been slower than expected (currently about 1/3 of the patients have been included), we wish to expand the study to more centers. The Minutes remain unchanged. The number of patients to be included remains unchanged (135). Karolinska continues to be the principal investigator for the study, performs randomization and continues to be involved in all treatment and follow-up of the patients. Attached are certificates from the following clinics:

Spine Clinic, Linköping University Hospital (local manager: Allan Abbott)
Orthopedic Clinic: County Hospital Ryhov, Jönköping (local manage: Anna Aspberg Ahl)
Orthopedic Clinic: Västerås Hospital (local manager: Björn Dahlman)
Orthopedic Clinic: Norrlands University Hospital, Umeå (local manager: Pawe ́l Grabowski)
Orthopedic Clinic: Sundvalss sjukhus (local ansvarig: Anna Grauers)
Orthopedic Clinic: Mälar Hospital Eskilstuna (local manager: Ingrid Ekenman/Ylva Bodén)

The amendment application fee of SEK 2000 has been paid to bank giro 794-5496, marked with "Dnr 2012/172-31/4 Gerdhem". A receipt of payment is attached.

Best regards

Paul Gerdhem
Orthopedics Clinic K54
Karolinska University Hospital, Huddinge
14186 Huddinge
0736-994409
paul.gerdhem@karolinska.se

The Regional Ethical Review Board in Stockholm
Received: 2015-06-09
Dnr:

Attachments:
Certificate from relevant business managers.

Copy for information: Karl-Äke Jansson, manager, orthopedic clinic, Karolinska University Hospital

This is to certify that this is an English translated version of the original Swedish document
[signature] Date 241209

APPROVAL Date 150610

Sigurd Vitols
Scientific secretary
The Regional Ethical Review Board in Stockholm

Regionala etikprövnings-
nämnden i Stockholm
Inkom: 2017 -04- 05
Dnr: 2017/609-32

Regionala etikprövningsnämnden i Stockholm

FE 289

171 77 Stockhlm

Studien "Skolios- resultat av behandling" godkändes av etikprövningsnämnden 2012-02-22 (Dnr 2012/172-31/4), med komplettering godkänd 2015-06-09 (Dnr 2015-1007/32).

Vi önskar att etikprövningsnämnden tar ställning till följande tillägg i projektet.

Deltagarna i studien har, innan inkludering, bedömts ha en idiopatisk skolios. Vissa av dessa har genomgått magnetkameraundersökning av nervstrukturerna i ryggen för att utesluta patologi som i sig skulle kunna orsaka ryggradsdeformiteten. Detta har man då gjort inom ramen för den pågående vården innan inklusion i studien, eller senare om snabb progress av skoliosen skett som föranlett byte av behandling.

För att säkerställa att det inte föreligger någon patologi i ryggen, samt för att säkerställa att skoliosen är av idiopatisk karaktär, önskar vi erbjuda magnetkameraundersökningar till alla patienter som inkluderats i studien, alltså även till de som inte genomgått en sådan inom ramen för den vanliga sjukvården. Magnetkameraundersökning av ryggen är en ofarlig, icke-invasiv undersökning, som tar cirka en timme att utföra.

Avgiften på 2000 kr har betalats in på bankgiro 794-5496 märkt med "Dnr 2012/172-31/4 Gerdhem". Kvitto på betalning bifogas.

Med vänliga hälsningar

Elias Diarbakerli
Ortopedkliniken K54
Karolinska universitetssjukhuset, Huddinge
14186 Huddinge
0704-240061
elias.diarbakerli@karolinska.se

Paul Gerdhem
Ortopedkliniken K54
Karolinska universitetssjukhuset, Huddinge
14186 Huddinge
0736-994409
paul.gerdhem@karolinska.se

GODKÄNNS Dat. 2017 -04- 13

Erik Näslund
Vetenskaplig sekreterare
Regionala etikprövningsnämnden
i Stockholm

The Regional Ethical Review Board in Stockholm
Received: 2017-04-15
Dnr: 2017/609-32

The Regional Ethical Review Board in Stockholm

FE289

171 77 Stockholm

The study "Scoliosis - results of treatment" was approved by the Ethical Review Board 2012-02-22 (Dnr 2012/172-31/4), with supplementation approved 2015-06-09 (Dnr 2015-1007/32).

We would like the Ethical Review Board to approve the following additions to the project.

The participants in the study have, prior to inclusion, been assessed to have idiopathic scoliosis. Some of these have undergone magnetic resonance imaging scans of the nerve structures in the spine to rule out pathology that could cause the spinal deformity. This has then been done within the framework of routine care prior to inclusion in the study, or later if rapid progression of the scoliosis has occurred that has prompted a change of treatment.

To ensure that there is no pathology in the back, and to ensure that the scoliosis is of an idiopathic nature, we wish to offer magnetic resonance imaging scans to all patients included in the study, i.e. even those who have not undergone such a scan within the framework of routine health care. Magnetic resonance imaging of the spine is a harmless, non-invasive examination, which takes about an hour to perform.

The amendment application fee of SEK 2000 has been paid to bank giro 794-5496 marked with "Dnr 2012/172-31/4 Gerdhem". A receipt of payment is attached.

Best regards

Elias Diarbakerli
Orthopedics Clinic K54
Karolinska University Hospital, Huddinge
14186 Huddinge
0704-240061

Paul Gerdhem
Orthopedics Clinic K54
Karolinska University Hospital, Huddinge
14186 Huddinge
0736-994409
paul.gerdhem@karolinska.se

APPROVAL Date 2017-04-13

Erik Näslund
Scientific secretary
The Regional Ethical Review Board
in Stockholm

This is to certify that this is an English translated version of the original Swedish document

.... Date 04-12-2024
