# Supplementary material for: Trunk rotation, spinal deformity and appearance, health-related quality of life, and treatment adherence: Secondary outcomes in a randomized controlled trial on conservative treatment for adolescent idiopathic scoliosis
Source: PLoS One. 2025 Apr 21;20(4):e0320581. doi: 10.1371/journal.pone.0320581 (PMC12011275; doi:10.1371/journal.pone.0320581)
Supplement: S4 File — (DOCX) [file pone.0320581.s004.docx]

**Human Participants Research Checklist**

***Complete the following if your study involved human participants or human participants’ data. These questions should be addressed for prospective and retrospective studies.***

1. Did you obtain ethics approval for this study?
   - If yes, please upload (file type “Other”) the original approval document you received from your ethics committee. If the original document is in another language, please also provide an English translation.

_X_ Uploaded ___ N/A

- - If you did not obtain ethical approval, please explain why this was not required below.

Full ethics application with approval in Swedish and English is provided in file S3 and S4 as a supplementary file to the manuscript.

1. If you prospectively recruited human participants for the study – for example, you conducted a clinical trial, distributed questionnaires, or obtained tissues, data or samples for the purposes of this study, please report in the Methods:
   1. the day, month and year of the **start and end** of the recruitment period for this study. Included in lines 116-117 of the manuscript. Consecutive recruitment took place from January 2013 to October 2018.
   2. whether participants provided informed consent, and if so, what type was obtained (for instance, written or verbal, and if verbal, how it was documented and witnessed). If your study included minors, state whether you obtained consent from parents or guardians. If the need for consent was waived by the ethics committee, please include this information. Included in line 222 of the manuscript as informed consent. Should be; informed written consent, for children and young people under the age of 15, consent was obtained from the parents/guardians.

_X_ Completed ___ N/A

1. If you are reporting a retrospective study of medical records or archived samples, please report in the Methods section:
2. the day, month and year when the data were accessed for research purposes
3. whether authors had access to information that could identify individual participants during or after data collection

___ Completed _X_ N/A
