# Supplementary material for: Trunk rotation, spinal deformity and appearance, health-related quality of life, and treatment adherence: Secondary outcomes in a randomized controlled trial on conservative treatment for adolescent idiopathic scoliosis
Source: PLoS One. 2025 Apr 21;20(4):e0320581. doi: 10.1371/journal.pone.0320581 (PMC12011275; doi:10.1371/journal.pone.0320581)
Supplement: S5 File — (DOCX) [file pone.0320581.s005.docx]

Response to the requested edits on our submission PONE-D-24-52488 - [EMID:5d2b78331e2bf2bf]

Trunk rotation, Spinal Deformity and Appearance, Health Related Quality of Life, and Treatment Adherence: Secondary Outcomes in a Randomized Controlled Trial on Conservative Treatment for Adolescent Idiopathic Scoliosis

1. During our evaluation of the documents provided, we noted that your ethics approval letter did not cover the entire date range for participant recruitment and data collection January 2013 to October 2018. Before we can proceed further with the submission, please provide the ethics approval extension document(s) for the study. If the document is in another language, please also provide an English translation.

Response: According to Swedish law, an ethics approval is valid until further notice provided that the research has commenced within two years from the date on which the decision on approval became final ([https://etikprovningsmyndigheten.se/faq/hur-lange-galler-ett-etikgodkannande/](https://eur01.safelinks.protection.outlook.com/?url=https%3A%2F%2Fetikprovningsmyndigheten.se%2Ffaq%2Fhur-lange-galler-ett-etikgodkannande%2F&data=05%7C02%7Cmarlene.dufvenberg%40liu.se%7C9479891f520f41da7e0e08dd182d79c4%7C913f18ec7f264c5fa816784fe9a58edd%7C0%7C0%7C638693305965975366%7CUnknown%7CTWFpbGZsb3d8eyJFbXB0eU1hcGkiOnRydWUsIlYiOiIwLjAuMDAwMCIsIlAiOiJXaW4zMiIsIkFOIjoiTWFpbCIsIldUIjoyfQ%3D%3D%7C0%7C%7C%7C&sdata=lJ3YKXCT0r8O6dk4OjKRrZF%2FqkHYOJFbdQKvQx%2BOs80%3D&reserved=0)). This means that the entire range of our participant recruitment and data collection falls within our approved ethics application. In addition to the approved original application Dnr 2012/172-31/4 in 2012 we have also approved amendments Dnr 2015/1007-32 in 2015, and Dnr 2017/609-32 in 2017. Added as supporting information: S3 Ethics documentation CONTRAIS in both Swedish and English.

2. Also, please report in the Methods section the day, month and year of the start and end of the recruitment period for this study in dd-mm-yyyy format . Please note that if this information is not included when your manuscript is resubmitted, it may be rejected

Response: We have now added the day, month and year of the start date (08-01-2013) and end date (23-10-2018) of the recruitment period for this study on page 6, row 117, in the Methods section of the manuscript.
