# Supplementary material for: Trunk rotation, spinal deformity and appearance, health-related quality of life, and treatment adherence: Secondary outcomes in a randomized controlled trial on conservative treatment for adolescent idiopathic scoliosis
Source: PLoS One. 2025 Apr 21;20(4):e0320581. doi: 10.1371/journal.pone.0320581 (PMC12011275; doi:10.1371/journal.pone.0320581)
Supplement: S6 File — (DOCX) [file pone.0320581.s006.docx]

Marlene Dufvenberg (Department of Health, Medicine and Caring Sciences, Unit of Physiotherapy, Linköping University, Linköping, Sweden).

Anastasios Charalampidis (Department of Clinical Science, Intervention and Technology (CLINTEC), Karolinska Institutet, Stockholm, Sweden).

Elias Diarbakerli (Department of Clinical Science, Intervention and Technology (CLINTEC), Karolinska Institutet, Stockholm, Sweden).

Anna Aspberg Ahl (Department of Orthopaedics, Ryhov Hospital, Jönköping, Sweden).

Birgitta Öberg (Department of Health, Medicine and Caring Sciences, Unit of Physiotherapy, Linköping University, Linköping, Sweden).

Hans Tropp (Department of Orthopaedics, Linköping University Hospital, Linköping, Sweden).

Hans Möller (Department of Clinical Science, Intervention and Technology (CLINTEC), Karolinska Institutet, Stockholm, Sweden).

Paul Gerdhem, Lead author for the study group, [paul.gerdhem@uu.se](mailto:paul.gerdhem@uu.se) (Department of Orthopedics and Hand Surgery, Uppsala University Hospital, Uppsala, Sweden).

Allan Abbott (Department of Health, Medicine and Caring Sciences, Unit of Physiotherapy, Linköping University, Linköping, Sweden).

Kourosh Jalalpour (Department of Reconstructive Orthopaedics, Karolinska University Hospital, Stockholm, Sweden).

Acke Ohlin (Clinical and Molecular Osteoporosis Unit, Department of Clinical Sciences, Malmö, Lund University, Lund, Sweden).

Anna Grauers (Sundsvall and Härnösand County Hospital, Sundsvall, Sweden).

Ylva Boden (Department of Orthopaedics, Linköping University Hospital, Linköping, Sweden).

Mats Hoffsten (Team Olmed, Stockholm, Sweden), Per Näsman (Center for Safety Research, KTH Royal Institute of Technology, Stockholm, Sweden).
