# Supplementary material for: Trunk rotation, spinal deformity and appearance, health-related quality of life, and treatment adherence: Secondary outcomes in a randomized controlled trial on conservative treatment for adolescent idiopathic scoliosis
Source: PLoS One. 2025 Apr 21;20(4):e0320581. doi: 10.1371/journal.pone.0320581 (PMC12011275; doi:10.1371/journal.pone.0320581)
Supplement: S7 File — (PDF) [file pone.0320581.s007.pdf]

Data set includes minimal anonymized data set necessary to replicate our study
-DB Table 2 Data document
-DB Table 3 Data document
-DB Table 4 Data document
-DB Table 5 Data document
-DB Table 6 Data document
-Table 2 Syntax
-Table 3 Syntax
-Table 4 Syntax
-Table 5 Syntax
-Table 6 Syntax

Detailed data, including personal data that directly or indirectly identifies patients, can only be provided upon reasonable request, provided that Swedish ethical approval has been obtained for such data. For this, we can refer to the following link. https://etikprovningsmyndigheten.se/wp-content/uploads/2024/05/Guide-to-the-ethical-review_webb.pdf .

The CONTRAIS Study Group
Paul Gerdhem, Lead author for the study group, paul.gerdhem@uu.se (Department of Orthopedics and Hand Surgery, Uppsala University Hospital, Uppsala, Sweden).
