# Supplementary material for: Trunk rotation, spinal deformity and appearance, health-related quality of life, and treatment adherence: Secondary outcomes in a randomized controlled trial on conservative treatment for adolescent idiopathic scoliosis
Source: PLoS One. 2025 Apr 21;20(4):e0320581. doi: 10.1371/journal.pone.0320581 (PMC12011275; doi:10.1371/journal.pone.0320581)
Supplement: Attachment — Submitted filename: evaluation-review.docx [file pone.0320581.s008.docx]

Thank you for writing the valuable article. Following I suggested some corrections on your article which hopefully make it better for your audiences.

**Introduction section**

Line 75: The verb “aims” came for “treatments”. If you want to say many kinds of treatment, you should use treatments with the related verb aim. Otherwise, you should use treatment with the verb aims.

**Conclusion section**

Please describe better what results can derive from your study. You mentioned a general description of the study. It is recommended to describe more detail and don not use the abbreviation to better conclude the message of your study.
